# Oncogenic roles of PRL-3 in FLT3-ITD induced acute myeloid leukaemia

**DOI:** 10.1002/emmm.201202183

**Published:** 2013-08-08

**Authors:** Jung Eun Park, Hiu Fung Yuen, Jian Biao Zhou, Abdul Qader O Al-aidaroos, Ke Guo, Peter J Valk, Shu Dong Zhang, Wee Joo Chng, Cheng William Hong, Ken Mills, Qi Zeng

**Affiliations:** 1 Institute of Molecular and Cell Biology, A*STAR (Agency for Science, Technology and Research)Singapore; 2 Cancer Science Institute of Singapore, National University of SingaporeSingapore; 3 Department of Hematology, Erasmus University Medical Center RotterdamRotterdam, The Netherlands; 4 Centre for Cancer Research and Cell Biology, Queen's University BelfastNorthern Ireland, UK; 5 Department of Haematology-Oncology, National University Cancer InstituteSingapore; 6 Department of Medicine, Yong Loo Lin School of Medicine, National University of SingaporeSingapore; 7 Cleveland Clinic Lerner College of MedicineCleveland, OH, USA; 8 Department of Biochemistry, Yong Loo Lin School of Medicine, National University of SingaporeSingapore

**Keywords:** acute myeloid leukaemia, antibody therapy, FLT3-ITD mutation, PRL-3, prognostic marker

## Abstract

FLT3-ITD mutations are prevalent mutations in acute myeloid leukaemia (AML). PRL-3, a metastasis-associated phosphatase, is a downstream target of FLT3-ITD. This study investigates the regulation and function of PRL-3 in leukaemia cell lines and AML patients associated with FLT3-ITD mutations. PRL-3 expression is upregulated by the FLT3-STAT5 signalling pathway in leukaemia cells, leading an activation of AP-1 transcription factors via ERK and JNK pathways. PRL-3-depleted AML cells showed a significant decrease in cell growth. Clinically, high PRL-3 mRNA expression was associated with FLT3-ITD mutations in four independent AML datasets with 1158 patients. Multivariable Cox-regression analysis on our Cohort 1 with 221 patients identified PRL-3 as a novel prognostic marker independent of other clinical parameters. Kaplan–Meier analysis showed high PRL-3 mRNA expression was significantly associated with poorer survival among 491 patients with normal karyotype. Targeting PRL-3 reversed the oncogenic effects in FLT3-ITD AML models *in vitro* and *in vivo*. Herein, we suggest that PRL-3 could serve as a prognostic marker to predict poorer survival and as a promising novel therapeutic target for AML patients.

## INTRODUCTION

Acute myeloid leukaemia (AML) is characterized by a block in differentiation and uncontrolled proliferation of malignant clones of immature myeloid cells (Lowenberg et al, 1999). Because of the high heterogeneity of acquired mutations occurring through unknown mechanisms, therapeutic approaches have limited efficacies and clinical outcomes of AML patients are poor (Sternberg & Licht, 2005). Activating mutations in fms-like tyrosine kinase-3 (FLT3) represent one of the more frequent genetic alterations in AML (Rockova et al, 2011), involving internal tandem duplication (ITD) in the juxtamembrane (JM) domain of FLT3 (Nakao et al, 1996). The constitutive activation of FLT3-ITD leads to elevated and sustained activation of multiple downstream signalling pathways, ultimately resulting in the transformation of haematopoietic cells to growth factor-independent proliferation (Mizuki et al, 2000). Due to pro-proliferative and anti-apoptotic roles in AML cells, activating mutations in FLT3 have been proposed as promising molecular targets for the treatment of AML. However, despite advances in drug discovery and our understanding of the molecular mechanism of FLT3 mutations, clinical trials with FLT3 inhibitors so far have shown limited success due to drug resistance and poor clinical response (Weisberg et al, 2010). This suggests that understanding of the underlying mechanism of FLT3 mutations may help in the development of better therapeutic strategies.

PRL-3, a phosphatase that we identified in 1998 (Zeng et al, 1998), was recently found as part of a core gene signature that is uniquely down-regulated by combination therapy of Linifanib (ABT-869, a FLT3 inhibitor) and suberoylanilide hydroxanic acid (SAHA, a histone deacetylase inhibitor) in AML cells (Zhou et al, 2011). Intriguingly, PRL-3 was recently reported as a possible downstream target of FLT3-ITD signalling, with a potential role in drug resistance of leukaemia cells (Zhou et al, 2011), indicating that PRL-3 expression levels could be an important factor contributing to the outcomes of the AML treatments. Furthermore, the Gene Expression Atlas (http://www.ebi.ac.uk/gxa/gene/ENSG00000184489) showed that the expression level of PRL-3 was the highest in chronic myeloid leukaemia (CML) among 950 human cancer cell lines covering 32 different types of cancers (Dataset code: E-MTAB-37), suggesting a potential role of PRL-3 in CML pathogenesis as well.

Previously, PRL-3 was first discovered to be specifically up-regulated in metastatic colorectal cancer cells (Saha et al, 2001) and subsequently reported to be associated with many other types of cancer metastasis such as breast, liver and gastric cancers (Bessette et al, 2008). Diverse roles of PRL-3 in cancer progression, including cell migration, invasion, proliferation, angiogenesis and metastasis, have been highlighted in recent reports that emphasize the importance of PRL-3 in tumourigenesis (Al-Aidaroos & Zeng, 2010; Liang et al, 2007). Increasingly, PRL-3 has emerged as a potentially useful biomarker for cancer prognosis, particularly the prediction of cancer metastasis (Matsukawa et al, 2010; Ren et al, 2009).

In this report, we investigated the role of PRL-3 in FLT3-ITD positive AML cells and patient samples. We describe the regulation of PRL-3 by a FLT3-Src-STAT5 signalling in AML cells. PRL-3 expression correlated positively with FLT3-ITD mutation in AML patients. PRL-3 overexpression was associated with the activation of c-Jun proto-oncogene and cell growth. Finally, we describe the clinical relationship between elevated PRL-3 expression and shorter overall survival in AML patients, and characterize elevated PRL-3 expression as an independent prognostic marker for AML. A critical role of PRL-3 in leukaemogenesis was revealed using PRL-3-targeted immunotherapy in a leukaemic mouse model, suggesting that PRL-3 could be a potential therapeutic target for AML.

## RESULTS

### PRL-3 is frequently up-regulated in AML patients with FLT3-ITD mutations

**Figure 1:**
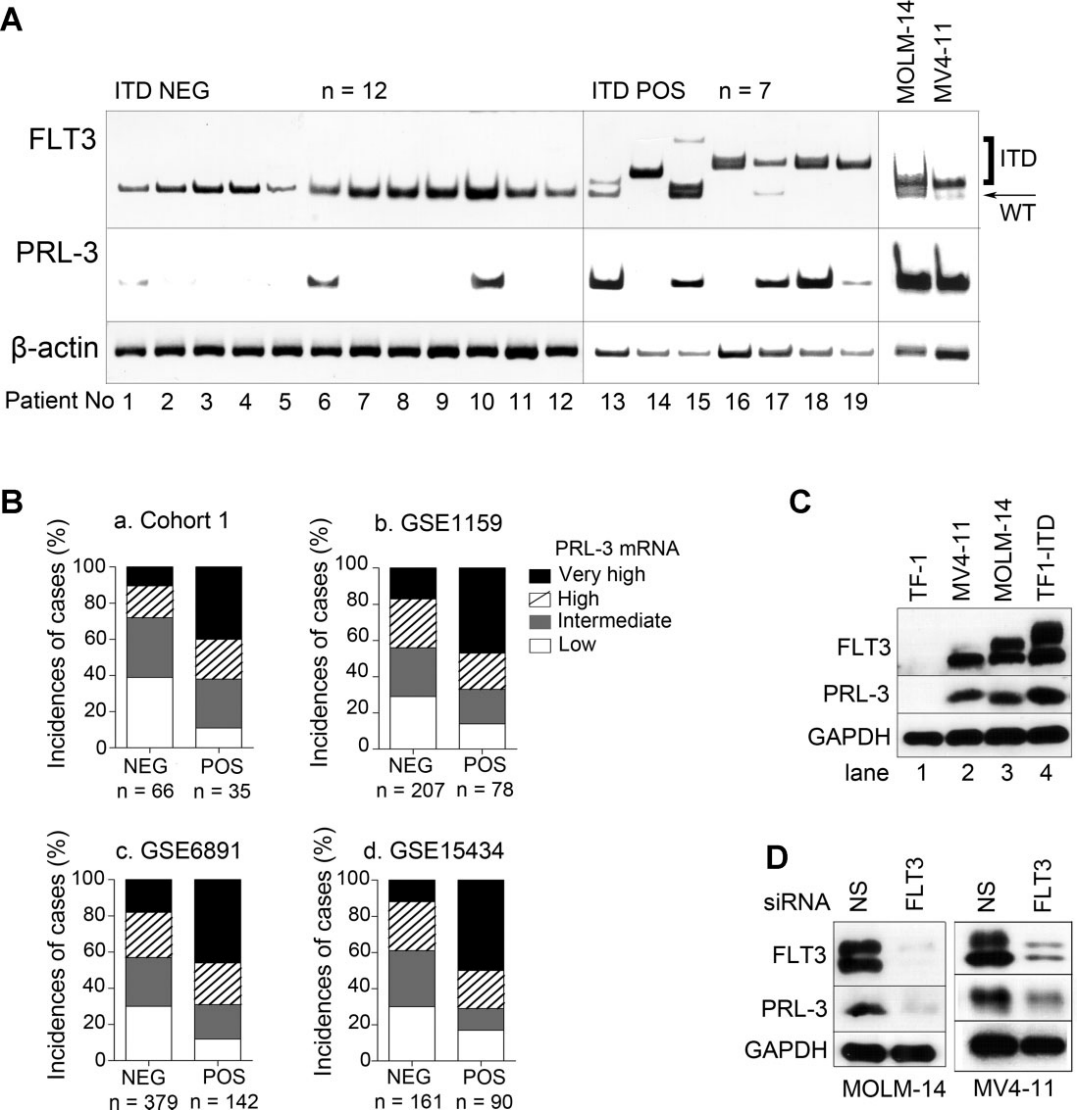
PRL-3 mRNA levels are elevated in FLT3-ITD-positive AML samples RT-PCR analysis of PRL-3 mRNA expression levels in 19 bone marrow samples from AML patients either negative (ITD NEG; *n* = 12) or positive (ITD POS; *n* = 7) for FLT3-ITD mutation. MOLM-14 and MV4-11 AML cell lines were used as FLT3-ITD positive controls. β-actin, loading control.(a–d) Microarray data analysis of PRL-3 mRNA levels in FLT-ITD-positive (POS) or FLT3-ITD-negative (NEG) patients in four independent patient cohorts (total *n* = 1158). (a) Cohort 1 AML patient with normal karyotype (*n* = 101, *p* = 0.001). (b) GSE1159 AML patient cohort (*n* = 285, *p* < 0.001). (c) GSE6891 AML patient cohort (*n* = 521, *p* < 0.001). (d) GSE15434 AML patient cohort (*n* = 251, *p* < 0.001). Statistical differences between ITD-POS and ITD-NEG patients were determined using Chi-square test. PRL-3 expression level is divided into four groups: very high, high, intermediate, low.Western blot analysis of PRL-3 protein levels in four AML cell lines.Western blot analysis of PRL-3 in MOLM-14 and MV4-11 cells upon siRNA-mediated knock-down of FLT3 expression. NS, control non-silencing siRNA. GAPDH, loading control.

To investigate a correlation between PRL-3 overexpression and FLT3-ITD mutations, 19 bone marrow samples from AML patients with or without FLT3-ITD mutations were analysed. The incidence of PRL-3 upregulation in AML was found to be highly associated with FLT3-ITD mutation (five out of seven cases, 71.4%), compared with only 3 out of 12 (25%) cases without FLT3-ITD mutation (Fig 1A). Similarly, high PRL-3 expression was observed in two FLT3-ITD positive cell lines (MOLM-14 and MV4-11; Fig 1A). Quantitative real-time PCR analysis of PRL-3 mRNA from the same patients supported that a higher PRL-3 mRNA expression was associated with AML patients with FLT3-ITD mutations (Supporting Information Fig S1). To extend this finding, we analysed our unpublished Belfast/MILE dataset (Cohort 1), consisting of total 221 AML patients. Among them, 101 patients with normal karyotype were used to analyse the relationship between PRL-3 expression level and FLT3-ITD mutation status. Only 10% of FLT3-ITD negative AML patients expressed ‘very highly’ PRL-3 (Chi-square test, *p* = 0.001), whereas over 40% of FLT3-ITD positive patients expressed ‘very highly’ PRL-3 (black block, Fig 1B, a). Our observation was further corroborated in three independent, publicly available AML patient datasets (GSE1159 *n* = 285, GSE6891 *n* = 521 and GSE15434 *n* = 251), where PRL-3 expression was consistently observed to be significantly higher in AML patients who were positive for FLT3-ITD mutation compared to those who were negative for FLT3-ITD mutations in three independent datasets (Fig 1B, b–d; Chi-square test; *p* < 0.001). In summary, our analysis of four separate AML patient cohorts show a strong association between FLT3-ITD mutations and high PRL-3 expression in a total of 1158 AML patients.

These results indicate that constitutive activation of FLT3 signalling might lead to PRL-3 overexpression in AML patients. To validate the clinical data, we either overexpressed or depleted FLT3-ITD in human myeloid leukaemia cell lines. Compared with TF-1 control cells (Fig 1C, lane 1), both MV4-11 and MOLM-14 cell lines harbouring endogenous FLT3-ITD mutations and TF-1 cell line over-expressing exogenous FLT3-ITD (TF1-ITD) had higher levels of PRL-3 (Fig 1C, lanes 2–4). In contrast, siRNA-mediated depletion of FLT3 expression in MOLM-14 and MV4-11 cells effectively suppressed PRL-3 expression (Fig 1D). Collectively, our results allude to a close relationship between FLT3-ITD mutation and elevated PRL-3 expression in AML cells.

### Constitutive activation of FLT3 enhances PRL-3 expression through Src-STAT5 signalling pathway

**Figure 2:**
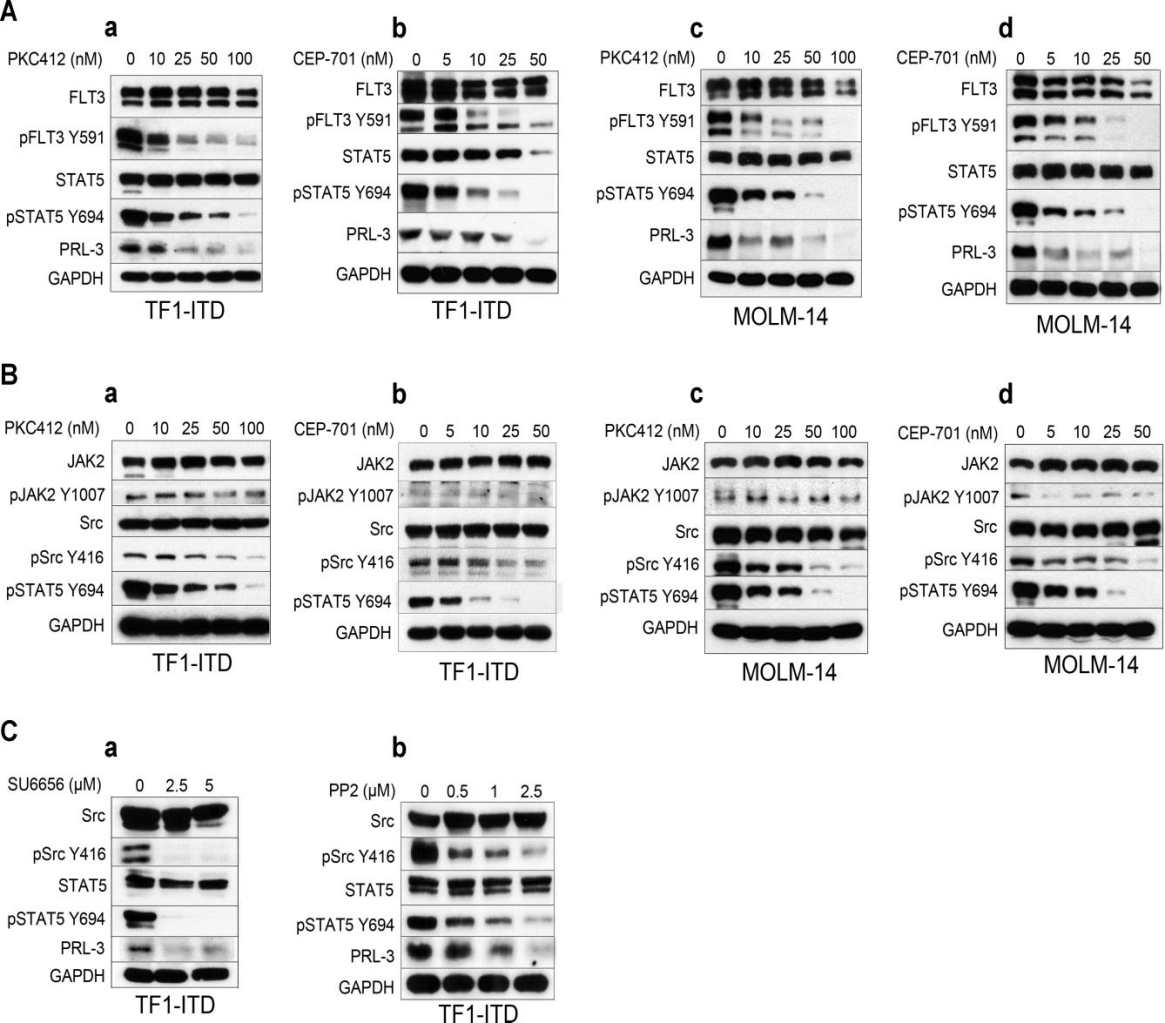
PRL-3 protein expression decreases upon FLT3 or Src inhibition in AML cell lines TF1-ITD and MOLM-14 cells were incubated with various concentrations of FLT3 inhibitors (PKC412, CEP-701) or Src inhibitors (SU6656, PP2) for 24 h. Whole cell lysates were subjected to Western blot analysis with indicated antibodies. GAPDH, loading control. (a–d) Western blot analysis of AML cells upon FLT3 inhibition. PKC412 and CEP-701 inhibited the phosphorylation of both FLT3 and STAT5 as well as PRL-3 protein levels in a dose-dependent manner in both TF1-ITD (a, b) and MOLM-14 (c, d) cells(a–d) Western blot analysis of AML cells upon FLT3 inhibition. PKC412 and CEP-701 inhibited the phosphorylation of Src, but not JAK, in a dose-dependent manner in both TF1-ITD (a, b) and MOLM-14 (c, d) cells.(a, b) Western blot analysis of AML cells upon Src inhibition. SU6656 (a) and PP2 (b) inhibited the phosphorylation of Src and STAT5 as well as PRL-3 protein levels in a dose-dependent manner in TF1-ITD cells.

To investigate if constitutively active FLT3 signalling was involved in upregulation of PRL-3 expression, we used FLT3 inhibitors to block FLT3 receptor activity and examined the downstream signalling molecules of FLT3-ITD mutation. Since STAT5 was known to be a critical downstream target of FLT3-ITD (Mizuki et al, 2000), we tested STAT5 expression level after treatment with FLT3-specific inhibitors; PKC412 or CEP-701 (Odgerel et al, 2007; Smith et al, 2004). The respective inhibitors reduced phosphorylation of FLT3 and STAT5 in a dose dependent manner and resulted in a corresponding decrease in PRL-3 protein levels in TF1-ITD and MOLM-14 cell lines (Fig 2A). We next examined whether FLT3-ITD-induced PRL-3 expression might be mediated by JAK or Src, two distinct upstream activators of STAT5 (Robinson et al, 2005; Spiekermann et al, 2003). After treatment with FLT3 inhibitors, both phospho- and total-JAK2 levels were not affected (Fig 2B), whereas the activated form of Src (pSrc Y416) was potently down-regulated after treatment. Importantly, Src inactivation closely corresponded with a decrease of STAT5 phosphorylation in a dose-dependent manner (Fig 2B). To investigate the role of Src-mediated phosphorylation of STAT5 in FLT3-ITD positive AML cells, AML cells were treated with two distinct Src kinase inhibitors, SU6656 and PP2 (Blake et al, 2000; Nam et al, 2002). Src inhibition reduced both STAT5 phosphorylation and PRL-3 expression levels (Fig 2C), revealing a correlation between Src-mediated STAT5 phosphorylation and PRL-3 expression.

### STAT5 is a potent transcriptional regulator of PRL-3 expression

**Figure 3:**
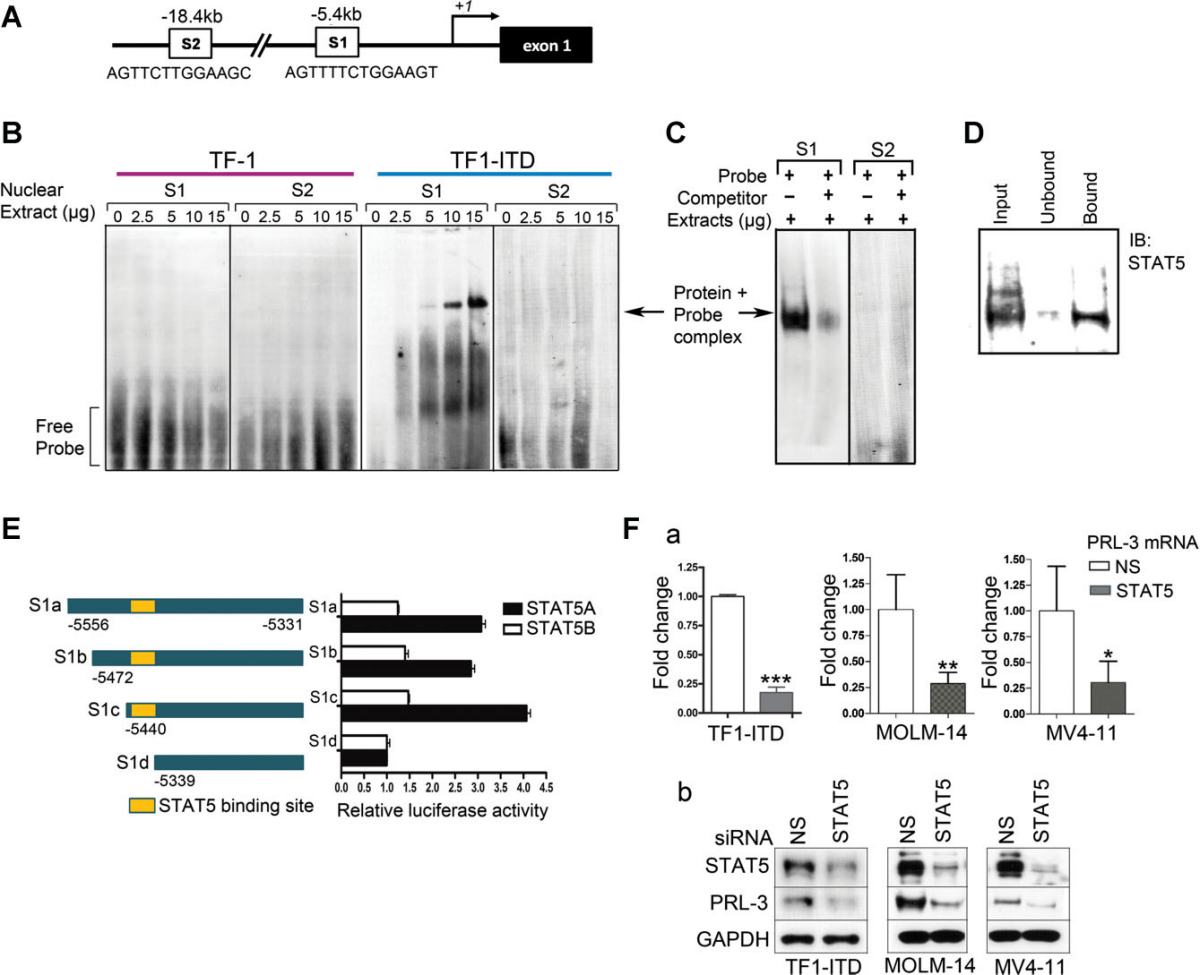
STAT5A is a direct transcriptional regulator of PRL-3 expression Two putative STAT5 binding sites (S1 and S2; DNA sequences illustrated) in a distal 5′-flanking region of PRL-3, as predicted by TRANSFAC.EMSA analysis using S1 and S2 biotinylated DNA probes (S1 and S2) incubated with nuclear extracts from either TF-1 or TF1-ITD cells. Arrow, shifted protein/probe complex.EMSA analysis as in (B) in the presence of 10-fold molar excess of unlabelled STAT5 competitor.Western blot analysis of streptavidin–agarose pull-down fractions (unbound or bound) using probe S1.Left panel: schematic diagram of a −5.4 kb upstream sequence of PRL-3 and its 5′-sequential deletion sequence with luciferase reporter vector (pGL3-S1a, S1b, S1c and S1d), respectively. Right panel: STAT5A or STAT5B expression vectors were co-transfected with PRL-3 luciferase reporter vector to TF-1 cells and luciferase activity measured. Error bars represent the mean ± SD from three independent experiments.PRL-3 expression is down-regulated upon siRNA-mediated STAT5 depletion in AML cells. NS, control non-silencing siRNA. (a) Quantitative real time PCR analysis of PRL-3 mRNA level after knock-down of STAT5 gene, normalized to GAPDH mRNA. Statistical differences between two groups were determined using Student's *t*-test (mean ± SD, *n* = 3). ***p < 0.001 (for TF1-ITD); **p = 0.011 (for MOLM-14); *p = 0.038 (for MV4-11). (b) Western blot analysis of PRL-3 protein level after knock-down of STAT5 gene. GAPDH, loading control.

To understand how PRL-3 could be up-regulated, the human PRL-3 promoter region was analysed by the Transcription Factor Database (TRANSFAC) to predict possible transcription factor binding sites (Wingender et al, 1996). The TRANSFAC program identified a number of putative transcription factors binding sites at the upstream promoter region of PRL-3, including two STAT5 consensus binding sequence TTCN(3)GAA (Seidel et al, 1995; Fig 3A). To evaluate the role of STAT5 as a transcriptional regulator of PRL-3, we designed two biotinylated probes, S1 and S2, corresponding to these STAT5 binding sequences and performed gel mobility shift assay (EMSA) using nuclear extracts from either TF-1 (PRL-3 non-expressing) or TF1-ITD (PRL-3 expressing) cells (Fig 1C, lanes 1, 4). Nuclear extracts from TF1-ITD cells exhibited a robust level of DNA binding activity specifically to probe S1 (−5.4 kb) but not to probe S2 (−18.4 kb) while nuclear extracts from parental TF-1 cells had no observable DNA binding activity with probe S1 or S2 (Fig 3B). Unlabelled competing oligonucleotides containing the STAT5 binding sequence could efficiently displace the labelled probe during the binding shift assay (Fig 3C). To further ensure the involvement of STAT5 in this protein/DNA complex, streptavidin–agarose pull-down assay was performed using biotinylated probe S1. Consistent with the EMSA result, Western blot analysis with STAT5 antibody confirmed that STAT5 was the transcription factor binding to the probe S1 in the complex (Fig 3D).

To further clarify the binding property of STAT5 to the upstream region of PRL-3 promoter, reporter assays were carried out using co-transfection of either STAT5A or STAT5B expression vector together with pGL3 luciferase vectors containing either the −5.4 kb upstream sequence of the PRL-3 promoter region or its sequential 5′-deletion constructs (S1a, S1b, S1c and S1d; Fig 3E, left panel). Similar protein expression levels of transfected STAT5A or STAT5B are shown in Supporting Information Fig S2. In TF-1 cells, when STAT5A expression vector was co-transfected with reporter constructs S1a–c, luciferase activities were increased three- to fourfold relative to the S1d deletion construct, which lacked of STAT5 binding site (Fig 3E, black columns). Interestingly, co-expression of STAT5B with the reporter constructs showed no significant increase in reporter activity (Fig 3E, open columns), suggesting that this activation could be specific for STAT5A but not for STAT5B. To support the role of STAT5 as a key transcription regulator of PRL-3, STAT5 was depleted by siRNA knock-down approach in three AML cell lines; TF1-ITD, MOLM-14 and MV4-11. Silencing of STAT5 attenuated PRL-3 mRNA (Fig 3F, a), consequently, decreased in PRL-3 protein expression levels (Fig 3F, b). These results further enforced the positive regulation of STAT5 on PRL-3 expression.

### Upregulation of PRL-3 activates AP-1 oncogenic transcription factor through ERK and JNK cascades

**Figure 4:**
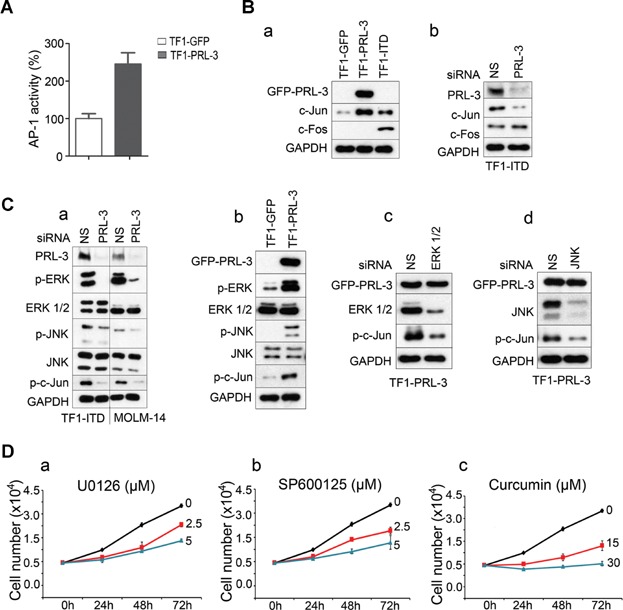
PRL-3 specifically activates c-Jun through ERK and JNK signalling pathways SEAP reporter assay results measuring AP-1 activity in TF-1 cells overexpressing GFP (TF1-GFP) or GFP-PRL-3 (TF1-PRL-3). Error bars represent the mean ± SD from three independent experiments.(a, b) PRL-3 specifically upregulates c-Jun but not c-Fos. (a) Western blot analysis of TF1-GFP, TF1-PRL-3 and TF1-ITD cells. (b) Western blot analysis after knock-down of endogenous PRL-3 in TF1-ITD cells. GAPDH, loading control. NS, control non-silencing siRNA.(a–d) PRL-3-mediated upregulation of c-Jun is dependent on ERK and JNK pathways. (a) Western blot analysis after knock-down of endogenous PRL-3 in TF1-ITD and MOLM-14 cells. (b) Western blot analysis of TF1-GFP and TF1-PRL-3 cells. (c, d) Western blot analysis after knock-down of ERK1/2 or JNK in TF1-PRL-3 cells. GAPDH, loading control. NS, control non-silencing siRNA.(a–c) MTS assay results reflecting numbers of viable TF1-PRL-3 cells after treatment with ERK-specific inhibitor (U0126), JNK-specific inhibitor (SP600125) or general AP-1 inhibitor (curcumin) for the various time points. Error bars represent the mean ± SD from three independent experiments.

PRL-3 has been reported to play important roles in tumour development (Guo et al, 2006; Matsukawa et al, 2010). Thus, we investigated the molecular consequences of PRL-3 overexpression on various oncogenic transcription factors, such as AP-1, a well-known transcription factor driving tumourigenesis (Eferl & Wagner, 2003). For this, we performed secreted alkaline phosphatase (SEAP) assay with pAP1-SEAP vector, which contains the SEAP reporter gene under the control of AP-1 promoter, using TF1-PRL-3 (TF-1 cells overexpressing GFP-PRL-3) and TF1-GFP control cells. As shown in Fig 4A, TF1-PRL-3 cells displayed a >2.5-fold increase in SEAP activity when compared to the TF1-GFP control cells, implying that PRL-3 could induce AP-1 expression. To investigate if this observation from TF-1 leukaemia cell line is applicable to solid tumour cell lines, two colorectal carcinoma cell lines, DLD-1 and HCT116, were examined. Consistently, overexpression of PRL-3 led to a >6.5-fold and >2.5-fold increase in AP-1 activity in DLD-1 cells and HCT116 cells, respectively (Supporting Information Fig S3).

To further identify the activated AP-1 complex, we performed Western blot analysis against c-Jun and c-Fos protein, the two key members of the AP-1 complex. Compared to TF1-GFP control cells, c-Jun was up-regulated in both TF1-PRL-3 and TF1-ITD cells while c-Fos was only detected in TF1-ITD but not in TF1-PRL-3 cells (Fig 4B, a), indicating that PRL-3 preferentially stimulates c-Jun but not c-Fos. This result was verified by knock-down of PRL-3 in TF1-ITD cells, which showed that the loss of PRL-3 reduced c-Jun (but not c-Fos) expression (Fig 4B, b). Since MAP kinases are actively involved in the regulation of AP-1 transcription factors (Zhang & Liu, 2002), we investigated whether induction of c-Jun might be a consequence of the activation of MAP kinases (MEK/ERK or JNK). Depletion of PRL-3 decreased the phosphorylation of JNK and ERK, leading to a subsequent loss of c-Jun phosphorylation in TF1-ITD and MOLM-14 cells (Fig 4C, a). In addition, overexpression of PRL-3 induced ERK and JNK phosphorylation in TF1-PRL-3 cells compared to TF1-GFP cells (Fig 4C, b). These results suggest that PRL-3 acts through ERK and/or JNK cascades to activate oncogenic c-Jun. To further confirm this, we knock-downed either ERK or JNK with respective siRNA in TF1-PRL-3 cells and the results showed that depletion of either ERK (Fig 4C, c) or JNK (Fig 4C, d) suppressed phosphorylation of c-Jun.

Since c-Jun is known to promote cell proliferation in various cancers (Hui et al, 2007; Zhang et al, 2007), we then investigated if activation of PRL-3-ERK/JNK-c-Jun pathway affect AML cell growth. TF1-PRL-3 cells treated with MEK-specific inhibitor (U0126, 5 µM) or JNK-specific inhibitor (SP600125, 5 µM) showed around 50% reduction in cell number compared to DMSO-treated control cells at 72 h (Fig 4D, a and b). In addition, treatment with 15 µM curcumin, a general inhibitor of AP-1 family (Balasubramanian & Eckert, 2007; Wang et al, 2009), decreased cell number to ∼50% of DMSO-treated cells at 72 h (Fig 4D, c).

### PRL-3 overexpression promotes cell growth and inhibits apoptosis

**Figure 5:**
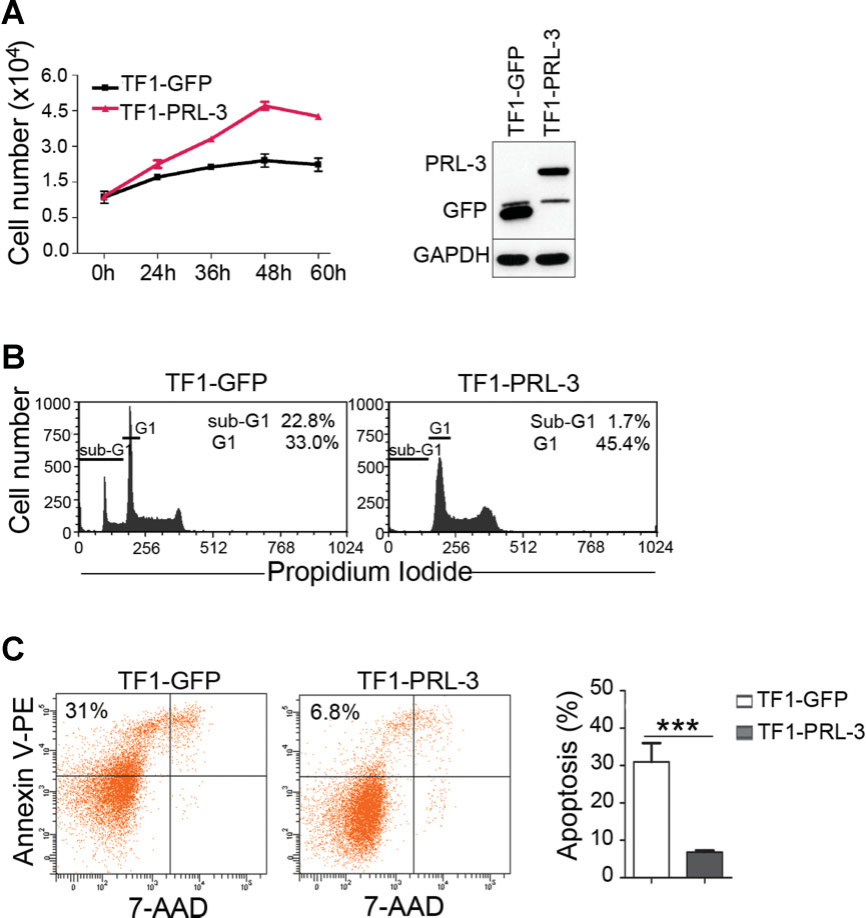
PRL-3 promotes growth and suppresses apoptosis of TF-1 leukaemia cells upon cytokine deprivation Right panel: MTS assay results reflecting numbers of viable TF1-GFP and TF1-PRL-3 cells after culture in the absence of cytokines for various durations. Error bars represent the mean ± SD from three independent experiments. Left panel: Western blot analysis of TF1-GFP and TF1-PRL-3 cells. GAPDH, loading control.Flow cytometry analysis of propidium iodide-stained TF1-GFP and TF1-PRL-3 after 48 h culture in the absence of cytokines. Note the difference in sub-G1 peak/population, reflective of apoptotic cells. Representative data from three independent experiments are shown.Left panel: flow cytometry analysis of annexin-V- and 7-AAD-stained TF1-GFP and TF1-PRL-3 after 48 h culture in the absence of cytokines. The percentage in the upper left quadrant indicates the fraction of annexin-V-positive apoptotic cells in the entire cell population analysed. Right panel: quantitation of annexin-V-positive apoptotic population in three independent experiments. Statistical differences between two groups were determined using Student's *t*-test (mean ± SD, *n* = 3, ***p = 0.0012).

To investigate the biological outcomes of PRL-3 overexpression, the gain of PRL-3 function in TF-1 cells was examined. TF-1 is a cytokine dependent cell line requiring supplementation of cytokines such as IL-3 or GM-CSF in culture media to sustain cell growth and survival (Lin et al, 2007). Without cytokine, TF-1-GFP vector control cells grow poorly (Fig 5A) and showed a 22.8% sub-G1 apoptotic population at 48 h time point (Fig 5B, left panel). However, TF-1 cells overexpressing PRL-3 (TF1-PRL-3 cells) became cytokine independent in term of cell growth and cell number increased to around twofold of TF1-GFP control cells at the same time point (Fig 5A). Furthermore, as presented in Fig 5B, TF1-PRL-3 cells had a much smaller sub-G1 apoptotic population (1.7%, Fig 5B, right panel) despite the lack of cytokine supplementation. To study anti-apoptotic activity of PRL-3 in the absence of cytokine supplementation, we performed annexin-V and 7-aminoactinomycin D (7-AAD) staining followed by fluorescence-activated cell sorting (FACS) analysis on TF1-GFP *versus* TF1-PRL-3 cell lines. More apoptotic population (31%) was observed in TF1-GFP cells than in TF1-PRL-3 cells (6.8%) after 48 h culture without cytokine supplement (Fig 5C), suggesting that PRL-3 might play an anti-apoptotic role and sustain the cell growth in TF1-PRL-3 AML cells.

### PRL-3 depletion reduces cell growth

**Figure 6:**
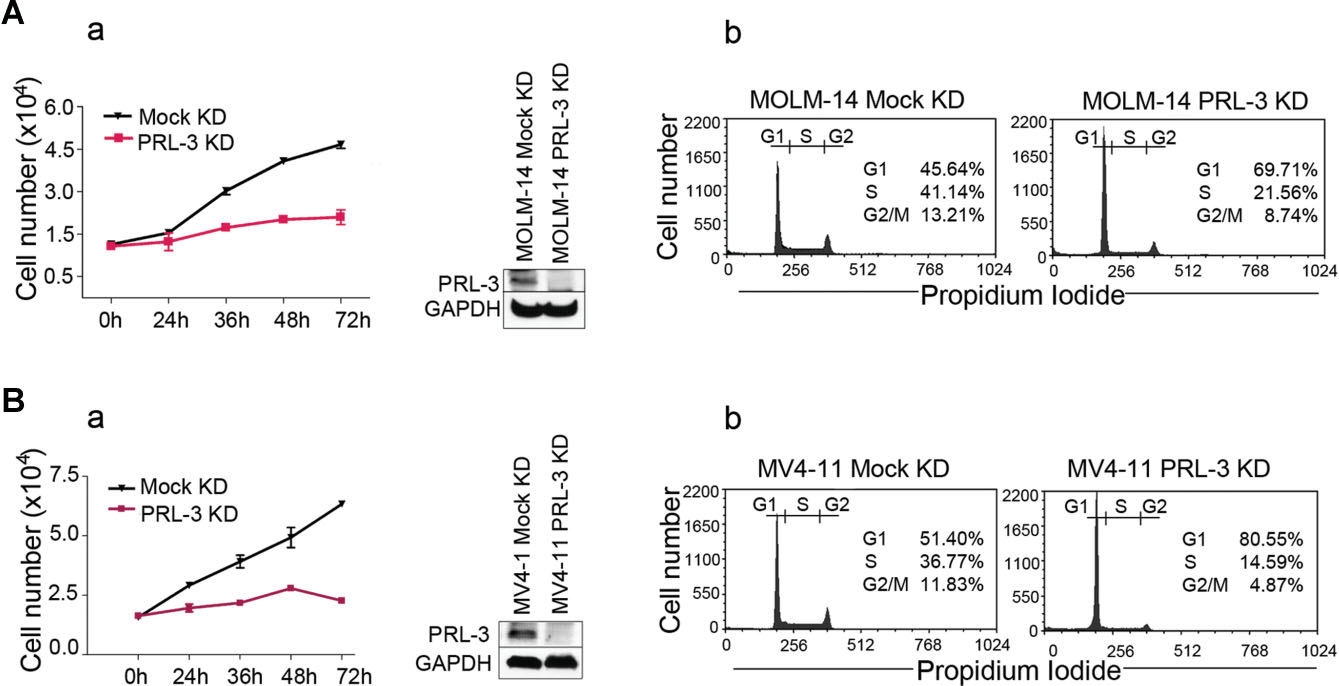
PRL-3 depletion inhibits the growth of FLT3-ITD-positive AML cells The growth of PRL-3-depleted MOLM-14 and MV4-11 FLT3-ITD-positive AML cells was analysed by MTS assay and flow cytometry. (a) Knock-down of PRL-3 decreased cell number in FLT3-ITD positive MOLM-14 cells (mean ± SD, *n* = 3). (b) Depletion of PRL-3 accumulated cells in G1 phase in MOLM-14 cells.(a) Knock-down of PRL-3 decreased cell number in FLT3-ITD positive MV4-11 cells (mean ± SD, *n* = 3). (b) Depletion of PRL-3 accumulated cells in G1 phase in MV4-11 cells. Representative data (right panel) from three independent experiments are shown.

To investigate the loss of PRL-3 function in AML cell lines, we knocked down of PRL-3 in two cytokine independent cell lines (MOLM-14 and MV4-11) that highly express both endogenous FLT3-ITD and PRL-3 (Fig 1C). After depletion of PRL-3, cell viability was assessed at various time points (Fig 6A, a and B, a). Interestingly, silencing of PRL-3 by siRNA resulted in reduced cell number by ∼64.5% in MOLM-14 cells and ∼66.7% in MV4-11 cells compared to their mock knock-down cells at 48 h. Furthermore, cell cycle analysis implied that the reduction in cell number in PRL-3-ablated cells correlated with increasing G1 and decreasing S phase populations (↑G1/S↓) in MOLM-14 and MV4-11 cell lines. The ratio of G1/S populations was 46.6%/41.1% in MOLM-14 mock knock-down cells, and became 69.7%/21.6% in MOLM-14 PRL-3 KD cells (Fig 6A, b). Similarly, the ratio of G1/S populations in MV4-11 mock knock-down cells shifted from 51.4%/36.8% to 80.5%/14.6% in MV4-11 PRL-3 KD cells (Fig 6B, b). Therefore, depletion of PRL-3 retards cells entering from G1 to S phase, implicating that PRL-3 may have roles in facilitating G1 to S phase transition to promote cell growth in both MOLM-14 and MV4-11 cell lines. However, depletion of PRL-3 did not affect on apoptosis as presented in cell cycle analysis (Fig 6A, b and B, b). Results showed that there were no observable increments of sub-G1 cell population after PRL-3 knock-down. It was further confirmed by apoptosis analysis with annexin-V and 7-AAD staining (Supporting Information Fig S4). FACS analysis showed that silencing of PRL-3 by siRNA did not show substantial increment of apoptotic population in both cell lines. These results imply that the role of PRL-3 is primarily in promoting G1-S transition in MOLM-14 and MV4-11 cytokine independent cells.

### PRL-3 antibody shows anti-tumour effect in mouse leukaemia model

**Figure 7:**
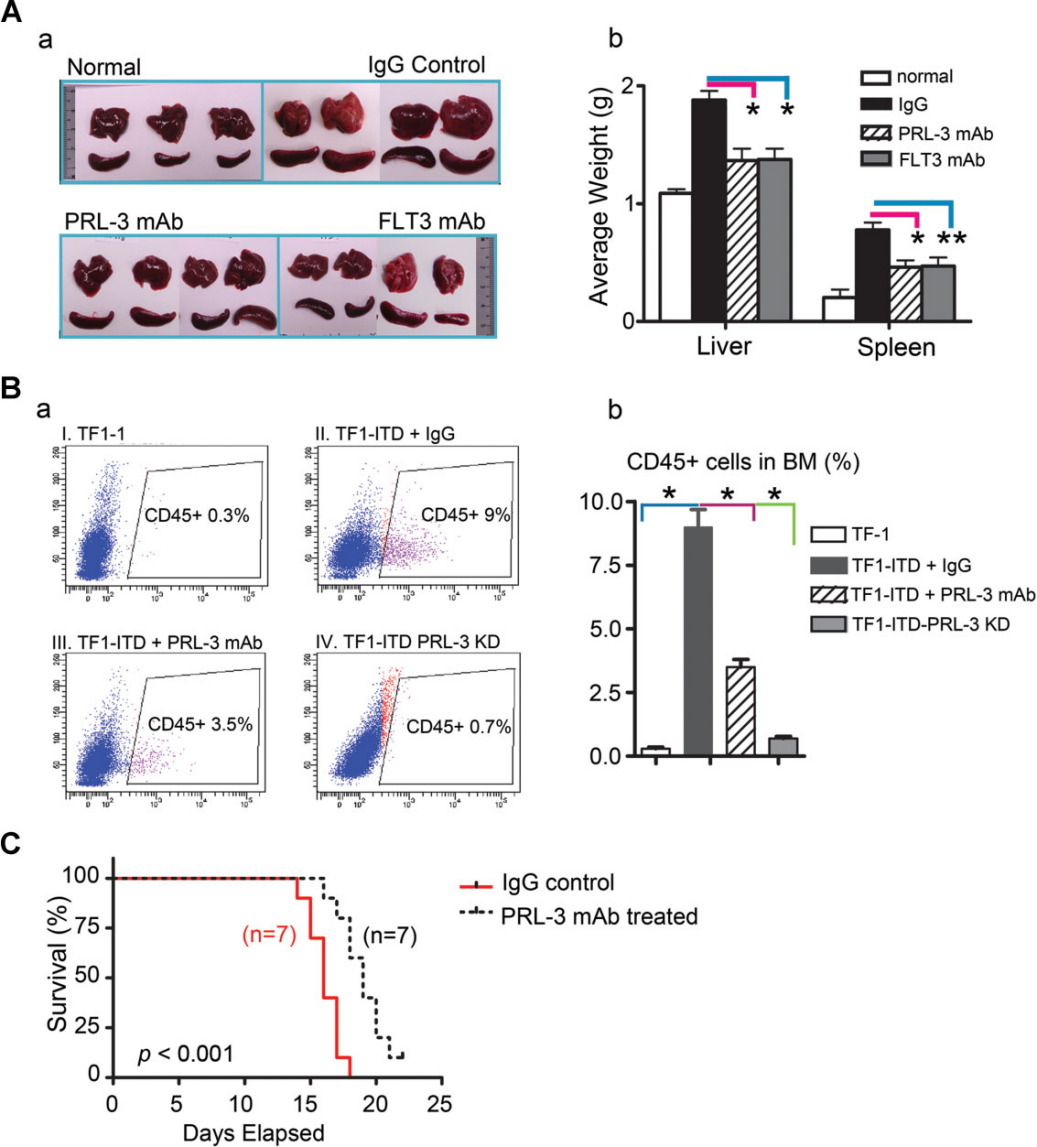
PRL-3 mAb exerts anti-tumour therapeutic effects in a mouse model of AML (a, b) Results of immunotherapy on liver and spleen sizes in a mouse model of AML. (a) Representative images of livers and spleens harvested from normal *nude* mice (upper left panel) or *nude* mice 12–14 days after i.v. injection of TF1-ITD cells together with bi-weekly i.v. administration of control IgG (upper right panel), PRL-3 mAb (lower left panel) or FLT3 mAb (lower right panel). (b) Quantitation of liver and spleen weights of mice as described in (a). Statistical differences between data groups were determined using Student's *t*-test from three independent experiments. *p < 0.001; **p = 0.00121.(a, b) Results of immunotherapy and PRL-3 knock-down on leukaemic infiltration in mouse bone marrow (BM) cells in a mouse model of AML. (a) BM cells from *nude* mice 12–14 days after i.v. injection of (I) TF1, TF1-ITD cells together with bi-weekly i.v. administration of (II) control IgG or (III) PRL-3 mAb (lower left panel) or (IV) TF1-ITD cells depleted of endogenous PRL-3 were analysed using flow cytometry analysis using the human-specific marker CD45+ to distinguish TF1 human-derived AML cells. Percentages indicate proportion of CD45+ cells in the BM population analysed. (b) Quantitation of CD45+ engrafted cells as described in (a). Statistical differences between two groups were determined using Student's *t*-test (mean ± SD, *n* = 5, *p < 0.001).Kaplan–Meier survival analysis of PRL-3 mAb-treated (*n* = 7) or control IgG-treated (*n* = 7) mice in the TF1-ITD leukaemia mouse model (*p* < 0.001).

Our results thus far showed FLT3 and PRL-3 could synergistically drive AML cell growth. Given that clinical trials with FLT3 inhibitors have shown primary or secondary drug resistance (Wiernik, 2010) and the implication of PRL-3 in AML drug resistance (Zhou et al, 2011), we herein attempted to develop an alternative strategy by using PRL-3 antibody to target PRL-3 (an intracellular phosphatase) expressing AML cells. We and others have demonstrated the feasibility of antibody therapy against intracellular oncoproteins for anticancer immunotherapy (Dao et al, 2013; Guo et al, 2011, 2012). To ascertain if the *in vitro* role of PRL-3 correlated with FLT3-ITD-driven AML tumour burden *in vivo*, we developed a leukaemia mouse model using the lateral tail vein injection of AML cells. PRL-3 monoclonal antibodies (mAb; Li et al, 2005) were subsequently used to target TF1-ITD AML cells which have elevated PRL-3 expression (Fig 1C, lane 4). Balb/c nude mice injected with TF1-ITD cells were divided into three treatment groups: (i) IgG antibody sham-treatment (IgG-treated, *n* = 11); (ii) PRL-3 mAb (PRL-3 mAb-treated, *n* = 11); or (iii) FLT3 mAb (FLT3 mAb-treated, *n* = 11). After bi-weekly administrations of IgG, PRL-3 or FLT3 mAbs over 12–14 days, PRL-3 mAb-treated mice showed a significant reduction of liver and spleen sizes (Fig 7A, a), indicative of reduced tumour burden. Liver and spleen weights were decreased to 72.8 and 59.3% of untreated (IgG control) group, respectively (*p* ≤ 0.001; Fig 7A, b). Notably, PRL-3 mAb treatment produced similar results to FLT3 mAb treatment (Fig 7A, a and b). Previously, FLT3 mAb treatment was demonstrated to have efficacy in an FLT3 leukaemia mouse model (Li et al, 2004). The current results corroborate a role of PRL-3 in FLT3-ITD-driven AML progression and indicate a novel use of PRL-3 antibody therapy to treat PRL-3 positive AML patients, in addition to other PRL-3-positive cancer types previously investigated (Guo et al, 2012).

To understand the effect of PRL-3 mAb in reducing leukaemia burden, we assessed the engraftment of these human leukaemic cells in mouse bone marrow. Twenty balb/c nude mice were divided into four groups (Fig 7B, I–IV, *n* = 5/group): Mice injected with (I) TF-1 cells; (II) TF1-ITD cells + IgG (IgG-treated); (III) TF1-ITD + PRL-3 mAb (PRL-3 mAb-treated); (IV) TF1-ITD PRL-3 KD (no treated). An antibody against the CD45 human specific haematopoietic cell surface marker (hCD45) was used to distinguish and identify engrafted human leukaemic cells from mouse host bone marrow cells by FACS analysis. Group I mice showed 0.3% of CD45-positive (hCD45+) cells engrafted in their bone marrows (Fig 7B, a, panel I). In contrast, group II mice showed 9% of cells in their bone marrows were hCD45+ (Fig 7B, a, panel II). Group III mice showed only 3.5% of cells being hCD45+ (Fig 7B, a, panel III), indicating that PRL-3 mAb treatment could reduce TF1-ITD cell infiltration. Group IV mice showed the effects of PRL-3 silencing on leukaemia development. We could detect only 0.7% of such hCD45+ cell in mouse bone marrow from group IV mice (Fig 7B, a, panel IV), suggesting that knock-down of PRL-3 was more effective than PRL-3 mAb treatment with regards to cancer cells engraftment in mouse bone marrow. The statistical significance of leukaemic infiltration in the different groups of mice is summarized in Fig 7B (b) (*p* < 0.001). Importantly, PRL-3 mAb therapy prolonged the survival rates for nude mice injected with TF1-ITD cells. Mice with a median survival of 19 days for PRL-3 mAb-treated but 16 days for control IgG-treated mice (Fig 7C; *p* < 0.001). Collectively, our results here demonstrate a significant benefit of PRL-3 immunotherapy in reducing FLT3-ITD AML cell engraftment in bone marrow and tumour burden, as well as in prolonging survival.

### PRL-3 expression in AML patients significantly associates with a shorter survival

**Figure 8:**
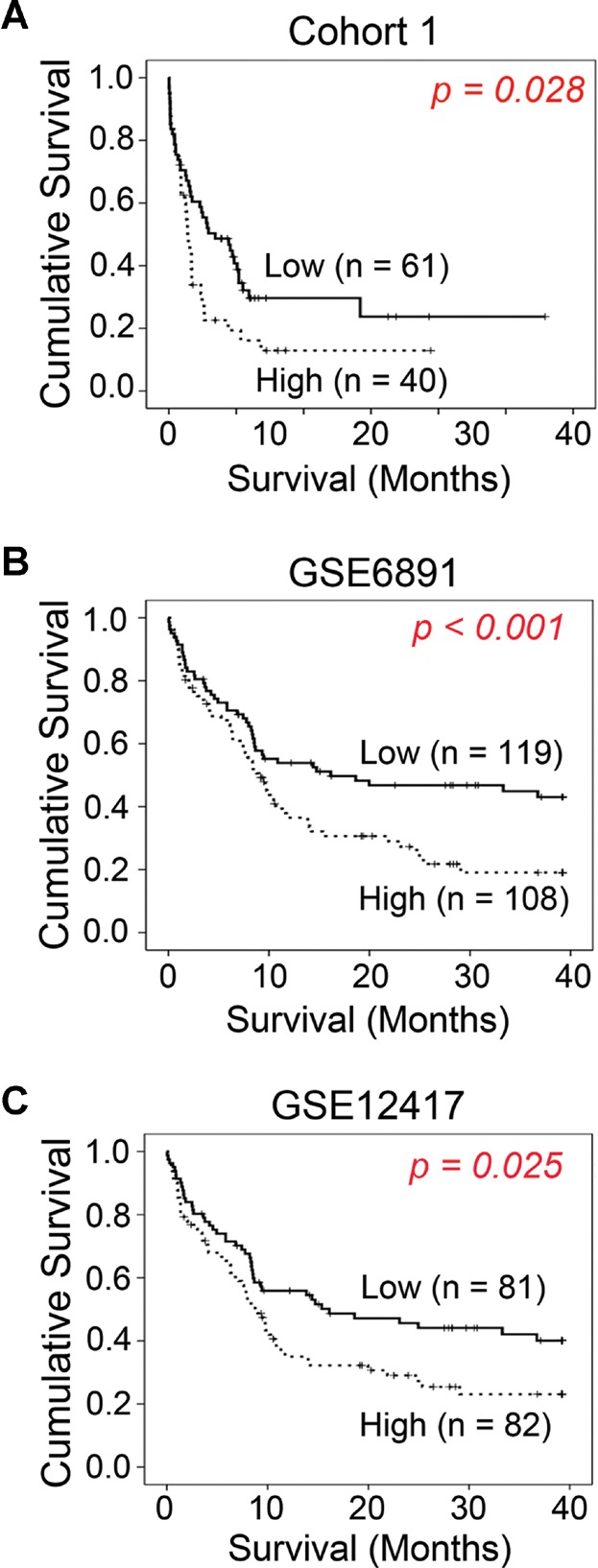
Elevated expression of PRL-3 correlates with a shorter survival in three independent AML patient cohorts **A–C.** Kaplan–Meier analysis of overall survival (OS) in normal karyotype AML patients for PRL-3 mRNA expression in (A) Cohort 1 AML patients, *n* = 101, (B) GSE6891, *n* = 227 and (C) GSE12417, *n* = 163. Statistically significant *p* values (using the log-rank test) are indicated in the figures.

To evaluate the clinical relevance and importance of PRL-3 expression in AML, the correlation between PRL-3 gene expression and the overall survival in AML patients was analysed using Cohort 1 (*n* = 221) and a publicly available dataset GSE12417 (*n* = 163) (Metzeler et al, 2008). By univariate Cox-regression analysis, high levels of PRL-3 expression were associated with a shorter survival in both Cohort 1 (HR = 1.327, 95% CI = 1.057–1.664, *p* = 0.015) and GSE12417 cohort (HR = 1.81, 95% CI = 1.20–2.74, *p* = 0.005). We noted that the prognostic value of PRL-3 was greater in AML patients with normal karyotype (*n* = 101) (HR = 1.576, 95% CI = 1.151–2.156, *p* = 0.005) than in AML patients with cytogenetic complications (*n* = 120) (HR = 1.298, 95% CI = 0.927–1.818, *p* = 0.129) in Cohort 1. We therefore focused on the relationship between PRL-3 and survival in such patients with normal karyotype by Kaplan–Meier survival analysis using the following three cohorts: (i) Cohort 1 (*n* = 101), (ii) GSE 6891 (*n* = 227) and (iii) GSE12417 (*n* = 163). In Cohort 1, high levels of PRL-3 expression were significantly associated with a shorter survival (mean survival time = 26 months, 95% CI = 13–40 months) compared to patients with low PRL-3 expression levels (mean survival time = 60 months, 95% CI = 39–81 months) in patients with normal karyotype (log-rank test, *n* = 101, *p* = 0.028; Fig 8A). In concordance, Kaplan–Meier survival analysis of AML patients with normal karyotype in the other two independent cohorts, GSE 6891 and GSE12417, also revealed that a high level of PRL-3 mRNA expression was significantly associated with a shorter survival time (log-rank test; *p* < 0.001 and *p* = 0.025, respectively; Fig 8B and C). Together, these results suggest that PRL-3 expression is associated with poorer overall survival in AML patients with a normal karyotype.

### Multivariable Cox-regression analysis reveals PRL-3 as an independent prognostic marker

To evaluate whether PRL-3 is an independent prognostic marker for survival in AML patients, multivariable Cox-regression was performed in Cohort 1 (*n* = 221) with parameters including sex, age, cytogenetic risk group, karyotype, FAB group, FLT3 mutation status, NPM mutation status and PRL-3 mRNA expression (Table 1). Importantly, high PRL-3 mRNA expression (*p* = 0.001, HR = 1.577, 95% CI = 1.199–2.073) was identified as an independent predictor for patient survival, in addition to age (*p* < 0.001), cytogenetic risk group (intermediate, *p* = 0.001; adverse, *p* < 0.001) and FLT3-ITD mutation (*p* = 0.001). Consistently, examination of the GSE6891 dataset (*n* = 521; Verhaak et al, 2009) using multivariable analysis likewise demonstrated that high PRL-3 expression or FLT3-ITD mutation were independent predictors for patient survival. In that dataset, we found that only a high level of PRL-3 expression (HR = 1.488, 95% CI = 1.194–1.855, *p* = 0.0004) or FLT3-ITD mutation (HR = 1.389, 95% CI = 1.094–1.764, *p* = 0.007) were shown to be independent predictors for patients survival. These consistent results from distinct datasets collectively indicate that a high level expression of PRL-3 is associated with poor survival, and highlight PRL-3 expression levels as an important and novel prognostic marker independent of other known clinically relevant prognostic markers for AML patients.

**Table 1:** Multivariate Cox-regression analysis reveal PRL-3 as an independent prognostic marker

	Cox-regression analysis of AML patient survival			
	
	Univariate analysis		Multivariate analysis	
		
Clinicopathological variables	Hazard ratio (95% CI)	*p*-Value	Hazard ratio (95% CI)	*p*-Value
Age (*n* = 221)	1.034 (1.022–1.046)	<0.0001	1.032 (1.019–1.045)	<0.0001
Sex				
Female (*n* = 112)	1	Reference		
Male (*n* = 109)	0.951 (0.681–1.326)	0.765		
Cytogenetic risk group				
Favourable (*n* = 63)	1	Reference	1	Reference
Intermediate (*n* = 142)	3.131 (1.950–5.026)	<0.0001	2.488 (1.458–4.244)	0.001
Adverse (*n* = 16)	5.528 (2.817–10.847)	<0.0001	4.431 (2.067–9.499)	<0.0001
Karyotype				
Normal (*n* = 101)	1	Reference		
Others (*n* = 120)	0.651 (0.465–0.911)	0.012		
FLT3 mutation status				
Normal (*n* = 141)	1	Reference	1	Reference
TKD (*n* = 29)	0.644 (0.358–1.156)	0.141	0.665 (0.355–1.243)	0.201
ITD (*n* = 50)	1.920 (1.322–2.789)	0.001	1.697 (1.303–2.896)	0.001
ITD/TKD (*n* = 1)	1.963 (0.272–14.155)	0.504	1.004 (0.133–7.590)	0.997
NPM mutation status				
Normal (*n* = 163)	1	Reference		
Mutant (*n* = 58)	1.319 (0.917–1.897)	0.136		
PRL-3 mRNA expression	1.306 (1.041–1.639)	0.021	1.577 (1.199–2.073)	0.001
FAB group (missing; *n* = 10)				
FAB group 1 (*n* = 50)	1	Reference		
FAB group 2 (*n* = 64)	0.736 (0.457–1.184)	0.206		
FAB group 3 (*n* = 12)	0.592 (0.231–1.519)	0.276		
FAB group 4 (*n* = 44)	0.662 (0.392–1.116)	0.122		
FAB group 5 (*n* = 35)	1.073 (0.640–1.799)	0.789		
FAB group 6 (*n* = 4)	1.504 (0.461–4.906)	0.499		
FAB group 7 (*n* = 2)	6.913 (1.598–29.903)	0.010		

PRL-3 acts as a novel prognostic marker in AML. By multivariate Cox-regression analysis using backward conditional stepwise method with a removal limit of *p* > 0.05, PRL-3 constituted one of the key independent predictors for poorer patient survival in our Cohort 1 (*n* = 221).

## DISCUSSION

In this study, we presented three major findings: (i) the molecular mechanism of PRL-3 overexpression in promoting AML cell growth *in vitro*, (ii) novel approach of using PRL-3 antibody as unconventional therapies to target PRL-3 expressing AML cells for reducing tumour burden in animal model and (iii) a clinical relationship between high PRL-3 expression and poorer survival in AML patients. Collectively, our findings suggest that PRL-3 could be a putative novel therapeutic target and a prognostic marker to predict poorer survival for AML patients with FLT3-ITD mutations.

FLT3 and its mutants have received much attention as therapeutic drug targets, due to their prominent roles in cell proliferation and differentiation of myeloblasts (Levis & Small, 2003). So far, several FLT3 selective inhibitors have been developed and examined in AML patients as single agents or in combination with chemotherapy (Wiernik, 2010). However, recent clinical trials with FLT3 inhibitors showed primary or secondary drug resistance and differential clinical outcomes (Weisberg et al, 2009). Thus, the discovery of critical downstream target genes of FLT3 mutation will be important for improved therapies. Herein, the demonstration of PRL-3 as a putative novel target for AML therapy is a timely and an important endeavour. Currently, only a handful of studies have addressed a possible link between PRL-3 expression and leukaemia (Fagerli et al, 2008; Zhou et al, 2011). Here we report that AML cells and patient samples with FLT3-ITD mutations have a high incidence of PRL-3 overexpression, an observation supported by the analysis of four separate AML patient cohorts in a total of 1158 patients. PRL-3 is shown to be a downstream target of FLT3-ITD mutation, with a FLT3-Src-STAT5 pathway regulating PRL-3 mRNA expression. Importantly, PRL-3 upregulation by FLT3-ITD mutations associated with cancer progression, a phenomenon potentially explained by the PRL-3-induced activation of oncogenic transcription factor c-Jun/AP-1. c-Jun is over-expressed in AML patients and contributes to a block in granulocyte differentiation and development of AML (Pulikkan et al, 2010; Rangatia et al, 2003), thus implicating an important role of AP-1 activation by PRL-3 in tumour development. In addition, treatment with MEK/JNK inhibitors (U0126, SP600125) or AP-1 inhibitor (curcumin) resulted in a decrease in PRL-3 driven-cell growth, suggesting that PRL-3 function is dependent on MEK/ERK and/or JNK signalling. Several reports have shown that PRL-3 could activate ERK through regulation of Rho family GTPase (Fiordalisi et al, 2006; Ming et al, 2009) or integrin β (Peng et al, 2009) in various solid cancer cells, but the detailed molecular mechanisms are not fully answered yet. In addition, it has been recently reported that PRLs (PRL-1, PRL-2 and PRL-3) can promote AP-1 activity and increase cell proliferation in non-small cell lung cancer cells (Hwang et al, 2011). Consistently, we demonstrate that PRL-3 played oncogenic roles in AML cell growth by promoting G1 to S phase transition in cell cycle as well as anti-apoptosis.

More importantly, we showed PRL-3 upregulation could contribute to AML progression, particularly in patients with normal karyotype, suggesting that PRL-3 was a viable therapeutic target for this group of patients, whose clinical outcomes to conventional therapies are highly heterogeneous (Baldus & Bullinger, 2008; Gaidzik & Dohner, 2008; Small, 2006). Moreover multivariable analysis validated PRL-3 expression as an independent prognostic marker in two distinct datasets (Cohort 1, GSE6891; Table 1). These results suggest that PRL-3 is a useful prognostic marker and a therapeutic target in AML patients.

Lastly, we demonstrated an unconventional antibody therapy approach to target intracellular PRL-3 oncoprotein for anti-AML therapy in mice (Fig 7). Antibodies are traditionally used to target extracellular (surface) proteins and have never been used to target intracellular proteins because antibodies are generally believed to be too large (∼150 kDa) to enter cells, leaving a large intracellular treasure of potential cancer-specific therapeutic targets untapped in terms of antibody therapy or vaccination. The possible mechanisms for how antibodies could target intracellular oncoproteins for anti-cancer were proposed in recent review articles (Ferrone, 2011; Guo et al, 2008, 2011; Hong & Zeng, 2012). Herein, this untraditional approach was further evaluated by performing PRL-3 mAb therapy in mice carrying tumours formed by TF1-ITD cells expressed both FLT3-ITD and PRL-3 proteins. Compared to control IgG-treated mice, mice treated with FLT3 mAb (targeting extracellular FLT3 receptor), or treated with PRL-3 mAb (targeting intracellular PRL-3) showed reduction in the sizes of spleen and liver, two enlarged organs commonly used for indicator of leukaemia burden. This result suggests a potential value of PRL-3 antibody therapy for AML patients associated with PRL-3 overexpression. Since FLT3 inhibition both alone and in combination with standard chemotherapy have proven clinical limitations, PRL-3 antibody therapy might provide a viable alternative treatment for AML patients with the FLT3-ITD mutation associated with PRL-3 overexpression. Such an antibody treatment might be particularly useful and specific to AML patients as leukaemia cells are easily accessible and are in direct contact with antibodies in their circulating system. The prospect of new therapeutic avenues by targeting PRL-3 in AML patients should be further explored.

## MATERIALS AND METHODS

### Cell lines and primary patient samples

TF-1 and MV4-11 cells were purchased from ATCC (American type culture collection, Manassas, VA). MOLM-14 cell line was obtained in house. TF-1 cells were cultured in RPMI 1640 (Invitrogen, Carlsbad, CA) supplemented with heat-inactivated 10% foetal bovine serum (Hyclone Laboratories, Inc., Logan, UT) and supplemented with 2 ng/ml human IL-3 (R & D system, Inc., Minneapolis, MN). TF1-ITD and TF1-PRL-3 cells were prepared as described previously (Kim et al, 2005; Zhou et al, 2011). Bone marrow (BM) blast cells were obtained from newly diagnosed AML patients with written informed consent from National University Hospital, Singapore. This study was approved by Institutional Review Board (IRB) of National University of Singapore.

### Chemicals and reagents

FLT3 inhibitor (PKC412), MEK inhibitor (U0126), p38 MAPK inhibitor (SB203580) and JNK inhibitor (SP600125) were purchased from LC Laboratories (Woburn, MA). FLT3 inhibitor (CEP-701) and Src kinase inhibitors (SU6656 and PP2) were purchased from Sigma (St. Louis, MO). Antibodies to FLT3, STAT5, JAK2, Src, ERK, c-Jun, pFLT3, pJAK2, pSTAT5, pSrc and p-c-Jun were purchased from Cell Signalling Technologies (Beverly, MA). GAPDH antibody was obtained from Millipore (Billerica, MA). Anti-CD45-APC and LightShift chemiluminescent EMSA kit were from Pierce Biotechnology, Inc. (Rockford, IL). Mouse anti-PRL3 antibody was from hybridoma clone 318 as reported previously (Li et al, 2005). SEAP reporter assay kit was purchased from Clontech (Palo Alto, CA) and Phospha-Light™ from Applied Biosystems (Bedford, MA).

### Detection of FLT3-ITD mutation and expression of PRL-3 by RT-PCR

Total RNA was extracted from AML patients' bone marrow cells using RNeasy minikit (Qiagen, Chatsworth, CA) according to the manufacturer's instructions. cDNA was synthesized from total RNA by reverse transcriptase III (Invitrogen) and amplified by PCR as described before (Quentmeier et al, 2003). The primer sets for RT-PCR were summarized; FLT3-ITD, 5′-GCAATTTAGGTATGAAAGCCAGC-3′ and 5′-CTTTCAGCATTTTGACGGCAACC-3′, PRL-3, 5′-GGGACTTCTCAGGTCGTGTC-3′ and 5′-AGCCCCGTACTTCTTCAGGT-3′, and the β-actin gene was 5'-GTGGGGCGCCCCAGGCACCA-3' and 5'-CTCCTTAATGTCACGCACGATTTC-3'. The PCR products were analysed on a 5% polyacrylamide gel, stained with ethidium bromide, and then visualized with GelDoc imager (BioRad, Inc., Hercules, CA).

### Quantitative real time PCR

Quantitative real time PCR (Q-RT-PCR) was used to measure the mRNA expression levels of PRL-3 at human BM samples and AML cell lines (ABI 7500 Fast Real Time PCR system). The cDNAs were served as template for Q-RT-PCR by using TaqMan® Universal PCR Master Mix kit (Applied Biosystems, Foster City, CA). Each 10 µl of quantitative PCR reaction mixture contained 5 µl of 2× TaqMan® Universal Master Mixture (Applied Biosystems), 4.5 µl of diluted cDNA mixture and 0.5 µl of gene specific probe. To standardize the quantification of the selected target genes, GAPDH served as internal controls and were quantified on the same plate as the target genes.

### Western blot analysis

Cells were lysed using modified RIPA buffer (50 mM Tris–HCl, 150 mM NaCl, 1% NP-40, pH 8.0, 1× protease inhibitor cocktail) and lysates were subjected to Western blotting with indicated primary antibodies. Proteins recognized by the antibodies were detected using the chemiluminescent detection kit (Pierce, Thermo Scientific, Rockford, IL).

### Transient transfection of siRNA or reporter vector

TF-1, TF1-ITD, MOLM-14 or MV4-11 cells were re-suspended at 2 × 10^6^ cells per 100 µl of appropriate Nucleofector kit solution (Amaxa Biosystems, Cologne, Germany) and were nucleofected with 2 µg of FLT3 SMARTpool siRNA duplexes (Dharmacon Research, Milipore), PRL-3 siRNA, Signal Silence STAT5 siRNA I/II (Cell Signalling Technologies), AP-1 SEAP reporter vector, ERK siRNA, JNK siRNA or non-silencing siRNA (Santa Cruz Biotechnology, CA). After nucleofection, the cells were immediately mixed with 500 µl of pre-warmed culture medium and transferred to culture plates for incubation. Samples were collected for protein extraction as described above.

### Electrophoretic mobility shift assay (EMSA)

The Transcription Factor Database (TRANSFAC) (Wingender et al, 1996) was used to predict possible transcription factor binding sites on human PRL-3 promoter region. Nuclear extracts were prepared using NE-PER nuclear protein extraction kit (Pierce, Thermo Scientific). For EMSA probes, 30 bp long, complementary sense and antisense strands of DNA oligonucleotides were annealed and diluted to 50 fmol/µl. DNA probes (50 fmol/µl) and nuclear extracts were mixed with EMSA buffer [50 mM MgCl_2_, 1% glycerol, 0.01% NP-40, 1 mM DTT and 0.1 mg/ml poly (dI-dC)] and incubated at room temperature for 30 min. In the competition reaction, unlabelled competitor was added in at least 10:1 molar excess over the biotinylated probe. Reaction mixtures were run on a native gel and visualised by LightShift chemiluminescent EMSA detection kit. The probe sequences (sense strand) used in this study include; S1, 5′-GGTGATGTTTTCTGGAAGTGTGGGT-3′, S2, 5′-CCATAAGTTCTTGGAAGCTGCGGCTT-3′ and STAT5 competitor sequence, 5′-AGATTTCTAGGAATTCAATCC-3′.

### Luciferase reporter assay

A −5.4 kb upstream region of PRL-3 (−5556 to −5331, S1a, numbered from a transcription initiation site) and its 5′-sequential deletion fragments (−5472 to −5331, −5440 to −5331 and −5399 to −5331; S1b, S1c and S1d, respectively) were subcloned into the pGL-Luc-basic vector. STAT5A and STAT5B expression vectors were purchased from OriGene Technologies, Inc. (Rockville, MD). TF-1 cells were seeded in 6-well plate and transfected with an expression vector of STAT5A or STAT5B along with 1.5 µg of an appropriate luciferase reporter construct by nucleofection method (Lonza Cologne AG, Switzerland). Luciferase assays were performed using Dual-Luciferase Reporter system (Promega, Madison, WI), in which relative firefly luciferase activities were calculated by normalizing transfection efficiency according to the renilla luciferase activities. The expression level of STAT5A or STAT5B was determined by Western blotting analysis.

### Secreted alkaline phosphatase (SEAP) assay

The AP-1 reporter vector (AP1-SEAP) was purchased from Clontech. TF1-GFP cells and TF1-PRL-3 cells were transfected with 200 ng of AP1-SEAP vector and incubated for 24 h. The culture supernatant was collected, heated at 65°C for 30 min, and assayed for alkaline phosphatase activity as follows; 30 µl of supernatant was incubated with 120 µl of assay buffer for 5 min, at which time 1:20 diluted CSPD substrate was added, and samples were read on a TECAN microplate reader (Maennedorf, Switzerland).

### Cell viability assay

AML cells (1 × 10^4^) were seeded into each well of 96-well tissue culture plates in 100 µl growth media and viable cells were measured after seeding with different inhibitors for 72 h using the CellTiter^96Aqueous^ cell proliferation assay kit (Promega). Briefly, an aliquot of 20 µl MTS mixture was added at the indicated time of assay and reactions were performed at 37°C for 2 h. And absorbance was read at 490 nm wavelength using TECAN microplate reader. After establishment of linear relationship between cell numbers and absorbance from each cell line, acquired absorbance converted to cell number.

**The paper explained**

PROBLEM:

The FLT3-ITD mutations are detected in 25–30% of AML patients and are associated with poor prognosis. Targeting FLT3-ITD mutations are a promising therapeutic approach for AML, however, clinical trials with FLT3 inhibitors have showed limited success. Insights into how FLT3 mutation leads to the disease will offer novel therapeutic opportunities.

RESULTS:

This study investigated the regulation and function of PRL-3, a metastasis-associated phosphatase, in leukaemia cell lines and AML patient samples associated with FLT3-ITD mutations. PRL-3 overexpression is mediated by the FLT3-Src-STAT5 signalling pathway in leukaemia cells, results in an activation of the AP-1 transcription factors via the ERK and JNK pathway. Depletion of PRL-3 attenuates cell growth and cell cycle progression *in vitro* whereas overexpression of PRL-3 enhances leukaemia development *in vivo*. PRL-3 antibody therapy reduced tumour burden in a leukaemia mouse model. The FLT3-ITD mutation was clinically associated with an increase in PRL-3 expression in four independent cohorts in a total of 1158 AML patients. Higher PRL-3 expression was significantly (*p* ≤ 0.001) associated with shorter survival in AML patients.

IMPACT:

The mechanistic findings on the FLT3-ITD-STAT5 signalling-mediated PRL-3 regulation unveiled the underlying mechanism of elevated PRL-3 expression that results in cell growth and tumour burden. Targeting PRL-3 reversed the oncogenic effects in FLT3-ITD AML models *in vitro* and *in vivo*, suggesting that PRL-3 is a promising therapeutic target. Performing multivariable Cox-regression in 221 AML patients of Cohort 1 identified PRL-3 as a novel prognostic marker independent of other clinical parameters.

### Cell cycle analysis

The DNA content of cultured cells was quantitated by staining with propidium iodide (PI) and analysed by flow cytometry (BDLSR11, Becton Dickinson, San Jose, CA). Briefly, cells were harvested with PBS and fixed with cold 70% ethanol at 4°C for 30 min. The cells were washed with PBS and then resuspended in 500 µl of PI staining solution and incubated for 30 min at room temperature. Samples were then examined and analysed for cell cycle phase (Modfit LT2.0, Becton Dickinson).

### Annexin-V and 7-aminoactinomycin D (7-AAD) staining

TF1-GFP and TF1-PRL-3 cells were harvested with PBS after 48 h culture in the lack of cytokine supplement. The cells were washed with PBS twice and incubated with annexin-V and 7-AAD staining solution for 30 min at room temperature. After staining, cells were subjected to FACS analysis.

### Cell line generation

To generate PRL-3 knock-down cell lines, pRS-PRL-3-shRNA (OriGene Technologies) was transfected into TF1-ITD cells. The resulting PRL-3-KD cell lines were selected with puromycin, and confirmed by Western blot analysis. TF1-ITD PRL-3-KD cells were used for mice injection.

### Anti-Leukaemic effects in mouse model

All animal studies as described previously (Guo et al, 2008) have been approved by Institutional Animal Care and Use Committee (IACUC). We followed the policies from the Animal Facility Center of The Agency for Science, Technology and Research (A*STAR), Singapore. Balb/c nude mice were obtained from Biological Resource Center (A*STAR, Singapore). Nude mice were intravenously injected with 1 × 10^6^ TF1-ITD cells. Three days later, mice were randomly divided into three treatment groups: injected twice weekly with IgG (sham treated, *n* = 11), PRL-3 mAb (PRL-3 treated, *n* = 11) and FLT3 mAb (FLT3 treated, *n* = 11). For survival study, mice treated with IgG (*n* = 7) or -treated with PRL-3 mAb (*n* = 7) were used and observed daily. Mice injected with TF-1 cells were used as controls for mice injected with TF1-ITD cells. Organs were isolated and inspected for macroscopic metastases at the end of the experiments (Guo et al, 2008).

### Human leukaemic cells engraftment analysis

Nude mice were intravenously injected with 1 × 10^6^ TF-1, TF1-ITD or TF1-ITD PRL-3 KD cells. Mice injected with TF1-ITD cells were divided into two groups and bi-weekly treated with control IgG or PRL-3 mAb. At the end of experiments, bone marrow cells were isolated and stained with human specific haematopoietic cell surface marker, CD45-APC antibody (Pierce, Thermo Scientific), and analysed by flow cytometry.

### Analysis of AML cancer patient microarray data

Details of all human AML patients datasets used in this study are summarized below and shown in Table 2. A total of five independent AML patient datasets were analyzed: (i) Our unpublished dataset (Cohort 1), analysed on Affymetrix U133Plus2 arrays from Belfast, UK; (ii) GEO-accessible GSE1159 dataset (Valk et al, 2004); (iii) GEO-accessible GSE6891 dataset (Verhaak et al, 2009); (iv) GEO-accessible GSE15434 dataset (Kohlmann et al, 2010) and (v) GEO-accessible GSE12417 dataset (Metzeler et al, 2008). Our summary showed the available information in each dataset. Datasets were pre-processed using R and Bioconductor for normalization. The median value was used as a cut-off point to differentiate high and low levels of PRL-3 (average of two PRL-3 probes; 206574 and 209695). Statistical analyses were performed using SPSS19.0 (IBM, Singapore). Correlation between PRL-3 expression and FLT3-ITD mutation status was analysed by Fisher exact test or Chi-square test where applicable. The association between PRL-3 expression and survival time was analysed by Kaplan–Meier analysis compared by log-rank test. *p* < 0.05 was considered significant. Cox-regression analysis with backward conditional stepwise selection with a removal limit of *p* > 0.05 was performed to identify independent predictors for AML patient survival.

**Table 2:** Details of all human AML patients datasets used in this study

				Information available				
						
No.	AML patient dataset	Total no. of AML patients with clinical dataset	No. of AML patients with normal karyotype	PRL-3 expression level	FLT3-ITD mutation status	Survival data	Reference	Figures or tables in this report using dataset information
1	Cohort 1	221	101	Y	Y	Y	In-house^b^	Figs 1 and 8, Table 1
2	GSE1159	285	NA^a^	Y	Y	N	Valk et al (2004)	Fig 1
3	GSE6891	521	227	Y	Y	Y	Verhaak et al (2009)	Figs 1 and 8
4	GSE15434	251	251	Y	Y	N	Kohlmann et al (2010)	Fig 1
5	GSE12417	163	163	Y	N	Y	Metzeler et al (2008)	Fig 8

Patient data was extracted and analysed in the leukaemia Gene Atlas platform (Hebestreit et al, 2012).

a No information available on cytogenetic profile of patients.

b Cohort 1 is a compilation of an in-house dataset and a MILE dataset from Belfast/MILE cohort.

## Author contributions

JEP designed and performed experiments, analysed data and wrote the paper; JBZ created the stable cell lines and contributed to discussion; HFY, AQOA-A revised the manuscript, analysed and interpreted the dataset; KG performed all mice experiments; WJC provided patient samples and AML cell lines; SZ analysed dataset; PJV provided and analysed dataset; CWH, discussed and edited the manuscript; KM provided and analysed dataset; QZ designed, supervised and revised the manuscript. All co-authors contributed to the revision of the manuscript.

## Supplementary material

As a service to our authors and readers, this journal provides supporting information supplied by the authors. Such materials are peer reviewed and may be re-organized for online delivery, but are not copy-edited or typeset. Technical support issues arising from supporting information (other than missing files) should be addressed to the authors.
