# Supplementary material for: Oncogenic roles of PRL-3 in FLT3-ITD induced acute myeloid leukaemia
Source: EMBO Mol Med. 2013 Aug 8;5(9):1351–66. doi: 10.1002/emmm.201202183 (PMC3799491; doi:10.1002/emmm.201202183)
Supplement: Supplementary file 1 [file emmm0005-1351-SD1.pdf]

EMBO Molecular Medicine

Oncogenic Roles of PRL-3 in FLT3-ITD Induced Acute Myeloid Leukemia

Jung Eun Park, Hiu Fung Yuen, Jianbiao Zhou, Abdul Qader O. Al-Aidaroos, Ke Guo, Peter J. Valk, Shu Dong Zhang, Wee Joo Chng, Cheng William Hong, Ken Mills and Qi Zeng

Corresponding author: Qi Zeng, Institute of Molecular and Cell Biology

Review timeline:

Submission date:	19 October 2012
Editorial Decision:	25 January 2013
Revision received:	26 April 2013
Editorial Decision:	11 June 2013
Revision received:	18 June 2013
Accepted:	20 June 2013

Transaction Report:

(Note: With the exception of the correction of typographical or spelling errors that could be a source of ambiguity, letters and reports are not edited. The original formatting of letters and referee reports may not be reflected in this compilation.)

Editor: Robert Buccione

1st Editorial Decision 25 January 2013

Thank you for the submission of your manuscript to EMBO Molecular Medicine. We are very sorry that it has taken so long to get back to you on your manuscript. In this case we experienced unusual difficulties in securing three willing and appropriate reviewers.

You will see that all three Reviewers are generally supportive of you work but raise significant issues that question the conclusiveness of the results and note several technical issues that preventing us from considering publication at this time. I will not dwell into much detail, as the evaluations are detailed and self-explanatory. I would like, however, to highlight a few main points.

Reviewer 1 is quite concerned about the results obtained using the PRL3 mAb in the AML mouse model, and suggests a number of crucial controls to validate the experiment. S/he notes additional issues that require your action.

Reviewer 2 also feels that some conclusions are invalid due to experimental flaws. Again, without going into detail I would mention his/her perplexities on the conclusion drawn from the immunoprecipitation experiments depicted in Fig. 2. Additionally, Reviewer 2 suggests a number of crucial experiments required to show that PRL-3 mediates up regulation of c-Jun through JNK/ERK. This Reviewer also lists other critical points that require your intervention.

Reviewer 3 feels that non-quantitative PCR measurements are not acceptable. I agree and would suggest you provide quantitative PCR data. S/he also has significant reservations on the description of the experiments described in Fig. 5 and mentions a number of other issues that your should act upon, including imrpoved figure legends.

While publication of the paper cannot be considered at this stage, we would be prepared to consider a suitably revised submission, with the understanding that the Reviewers' concerns must be fully addressed with additional experimental data where appropriate and that acceptance of the manuscript will entail a second round of review.

Since the required revision in this case appears to require a significant amount of time, additional work and experimentation and might be technically challenging, I would therefore understand if you chose to rather seek publication elsewhere at this stage. Should you do so, we would welcome a message to this effect.

Please note that it is EMBO Molecular Medicine policy to allow a single round of revision only and that, therefore, acceptance or rejection of the manuscript will depend on the completeness of your responses included in the next, final version of the manuscript.

As you know, EMBO Molecular Medicine has a "scooping protection" policy, whereby similar findings that are published by others during review or revision are not a criterion for rejection. However, I do ask you to get in touch with us after three months if you have not completed your revision, to update us on the status. Please also contact us as soon as possible if similar work is published elsewhere.

***** Reviewer's comments *****

Referee #1 (Comments on Novelty/Model System):

Technical quality is high, albeit the data in Fig. 6 are counterintuitive and require many additional controls. Medical impact is medium. Whereas identification of PRL3 as prognostic marker is convincing (high medical impact), the data using PRL3-specific mAbs to target AML cells in nude mice is not convincing (low impact).

Referee #1 (Remarks):

Park et al. investigated the role of PRL3 in AML in relation to the FLT3-ITD mutation. PRL3 expression is upregulated in a significant proportion of FLT3-ITD positive AML patients and cell lines, which is dependent on FLT3 and Src, but not JAK activity. STAT5 is activated concomitantly and PRL3 promoter activity is regulated by STAT5. PRL3 expression in turn is correlated with Jun/AP1 activation and enhanced proliferation. Injection of PRL-3 mAb in mice with engrafted TF-ITD tumors led to a reduction in TF-ITD tumors. Finally, PRL3 expression was identified as a novel prognostic marker independent of other clinical parameters.

This is an interesting paper that reveals the signaling pathway that results in elevated PRL3 expression in FLT3-ITD-positive tumor cells and it describes the identification of PRL3 as a prognostic marker for AML.

Points

1. Fig. 4. Overexpression of PRL3 leads to activation of AP1 in a MEK and JNK dependent manner. It is not evident how PRL3 would mediate this effect. This should be discussed.

2. The results using PRL3 mAb in the nude TF1-ITD mouse model for AML are puzzling. How can antibodies directed against an intracellular protein affect growth of the target cells in the tumor. The authors apparently have published this unconventional antibody therapy before (discussion, p.14). These data warrant rigorous controls, as these results are not only unconventional, but also counterintuitive. Does PRL3 mAb treatment only affect TF1-ITD cells or other tumors as well? For instance, are FLT3-ITD-independent AML cells that do not express elevated levels of PRL3 also affected by PRL3 mAb? What is the underlying working mechanism of the mAb treatment?

Referee #2 (Comments on Novelty/Model System):

Summary of the major findings:

In this study the authors analyze the correlation between the overexpression of PRL-3, a previously identified metastasis-associated tyrosine phosphatase, and FLT3-ITD in AML. By statistical analysis of AML patient data sets the authors show a link between PRL-3 overexpression and FLT3-ITD mutation.

Experiments with AML cell lines show that the constitutive activation of FLT3 enhances PRL-3 expression through the Src-STAT5 signaling pathway and that the up-regulation of PRL-3 induces the expression of c-Jun. However, the study does not provide convincing evidence that PRL-3 acts through a MAPK cascade. More experiments are needed to prove this point.

The authors also analyze effects of PRL-3 on cell proliferation and apoptosis and show that PRL-3 promotes cell proliferation in MTS assays while reducing the number of cells in apoptotic fraction in FACS analysis.

The in vivo role of PRL-3 for FLT3-ITD-driven tumor formation was analyzed in a mouse leukemia model by showing that treatment with as monoclonal PRL-3 antibody has anti-tumor effects. The PRL-3 mAb reduces spleen and liver size in mice injected with AML cells comparable to the treatment with FLT3 mAb and reduces AML cell infiltration into the bone marrow in xenograft models. The authors thus demonstrate a potential benefit of PRL-3 immunotherapy.

Additionally, statistical evidence is provided showing a correlation of PRL-3 expression with shorter survival in AML patients and revealing PRL-3 as an independent prognostics marker.

Overall, the present study follows up on findings of a previous publication (Zhou et al. 2011), which first showed the correlation between FLT3-ITD mutation and PRL-3 overexpression. Zhou et al. also show that treatment of MOLM14 cells with a PRL-3 inhibitor increases apoptosis which the present study also shows using FACS analysis in figure 5B. The present study presents a significant amount of new data. However, the study has some major flaws. The presented data was not convincing in several cases and some of the conclusions drawn were invalid. The below listed experiments should be repeated or altered as suggested. Especially the study of the in vivo functions of PRL-3 and the potential use of a PRL-3 mAb as a treatment for FLT3-ITD positive patients are of interest from a medical point of view and would merit re-submission of the manuscript.

Comments:

Major issues:

1.

The regulation of PRL-3 by FLT-3 analyzed in figure 2A is not convincing in the MOLM-14 cells, as the down-regulation of PRL3 is hardly visible in the western blot analysis. This experiment should be repeated in order to prove that FLT3 activity regulates PRL-3 expression.

2.

2.1.

In figure 2D the authors analyze the interaction between Src and FLT3 by immunoprecipitation studies in TFI-ITD cells and state that Src an FLT3 do not interact. However, there seems to be some residual Src binding at time point 0, but this hard to tell as the blots in the figure are of poor

quality. Negative controls for co-immunoprecipitation experiments and western blots showing protein levels in cellular lysates should be included in the figures.

2.2

The conclusion drawn from the co-immuniprecipitation experiment in figure 2D is not valid. If as claimed Src bridged the interaction between FLT3 and STAT5 then an IP:FLT3 should still pull down Src and STAT5 from cellular lysates. Indirect interactions can be seen in IPs from cellular lysates.

The authors should conduct pull-down experiments using purified recombinant proteins in order to analyze whether the interaction between FLT3 and STAT5 is direct or indirect.

3.

In figure 4 the authors make a good case of showing that the expression of PRL-3 induces expression of c-jun. However, they state that PRL-3 acts through a JNK/ERK signaling cascade. They do not provide any prove for this hypothesis. They show that inhibitors of the MAPK JNK and MEK as well as siRNA targeting JNK and ERK result in a decrease in c-Jun phosphorylation. This is to be expected as both JNK and MEK/ERK have been shown to be upstream of c-Jun. The relationship with PRL-3 remains obscure as experiments merely show that c-Jun phosphorylation is mediated by ERK and JNK, which is no new finding.

In order to show that PRL-3 mediates up-regulation of c-Jun through JNK/ERK the authors should conduct the following experiments:

- AML cell lines should be depleted of PRL-3 by siRNA and it should be shown that phosphorylation of JNK/ERK is reduced, either by western blotting or by an in vitro kinase assay.

- Over-expression of PRL-3 in PRL-3 negative cell line (i.e. TFI cells) should induce phosphorylation of JNK and ERK

4.

A more specific JNK inhibitor in should be used in figure 4D in order to prove that the growth advantage of PRL-3 expressing cells relies on activation of the JNK pathway as curcumin effects multiple signaling pathways

The conclusion drawn from this experiment is not valid. The authors suggest that PRL-3 is up-stream of c-Jun. However, The experiments presented in 4A and B already show this. The only new result is that curcumin does not affect PRL-3 levels.

Minor issues:

1.

Western blot analysis of phospho-FLT3 proving that the FLT3 inhibitors used in figure 2 are either missing or of poor quality. Expression levels of FLT3 in cellular lysates show a high variability at the analyzed time points.

2.

A c-fos western blot should be included in figure 4b.

3.

In figure 1 a PRL-3 band clearly visible in FLT3-ITD negative first sample. Thus, 3 out of 12 (25%) FLT3-ITD negative patients appear to show PRL-3 expression. The FLT3 band on far right of FLT3-ITD positive sample appears very faint. In order to strengthen the correlation between FLT3-ITD mutation and PRL-3 expression, an equal number of FLT3-ITD positive and negative AML patient samples should be analyzed.

4

The effects of PRL-3 overexpression or depletion on AML cells appears marginal in the FACS analysis in figure 5B. The study would benefit from a different apoptosis assay to prove an anti-apoptotic role of PRL-3 more convincingly.

Referee #3 (Comments on Novelty/Model System):

Important for the reasons given below, along with suggestions for a better model (in the future; these models are not sufficiently developed yet, in my opinion.

Referee #3 (Remarks):

Summary: The treatment of AML (with the exception of APL) has not changed much in the past 40 years, certainly not since this reviewer last cared for an AML patient in 1984. Therefore, it is critical that we develop novel therapies, and PRL3 is certainly an interesting candidate. Here the authors demonstrate an association between FLT3-ITD and PRL3, investigate the mechanism connecting the two, and demonstrate therapeutic efficacy of PRL3 antibodies. The findings are important in that we desperately need new therapeutic options in AML.

Suggestions for improvement of the manuscript:

There are a number of "minor" but still important corrections that need to be addressed:

- Fig. 1A. I do not understand why anyone does non-quantitative PCR anymore. If the RNA or samples are available, please repeat in a quantitative fashion. If not available, do it next time!

- The data supporting the role of STAT5 is much stronger than that of SRC; the authors should soften their statements in the results and discussion.

- Fig. 3. It would be desirable (but not absolutely required) to add a STAT5 antibody supershift to the EMSA, and a ChIP assay demonstrating STAT5 binding in cells. The streptavidin-agarose bead assay is a nice addendum to the EMSA.

- Through the manuscript, the authors refer to proliferation (references to Pulikkan and Rangatia page 8, their own Fig. 4C, etc). I believe they are using this term incorrectly, like most of the world. If I understand Fig. 4C correctly, they are measuring cell number, not proliferation. Please correct throughout the text (and please point out to colleagues that assays of cell number are NOT assays of cell proliferation!).

- Again, in Fig. 5, the authors have completely confused their assays, mixing up cell number, proliferation, and apoptosis. They need to be VERY precise in measuring cell number, proliferation, and apoptosis and be very careful in how they state the results and discussion. It is totally mixed up here, but it can all be easily fixed by being more precise in the labeling and language used. They need to restrain their interpretation of the relative effects of proliferation and apoptosis on cell number unless they perform more assays specific for proliferation and apoptosis.

- Fig. 6. It would be much more desirable to perform these experiments in a better model of FLT3-ITD AML than intravenous injection of transformed cell lines, but the published models using FLT3-ITD alone (either retroviral or knock-in) are not robust. Newer models combining FLT3-ITD with other mutations look more promising, and it will be of great interest to test these models in the future with PRL3 antibody by itself and combined with FLT3-ITD inhibitors.

- Fig. 7. I never understand the value of investigating the prognostic value unless the therapy is related to the gene of interest. Almost all these patients are treated with anthracycline/AraC, so unless response to these drugs is related to PRL3, I do not know how to interpret the data. When PRL3 antibody is used clinically, it would make more sense. This is not natural history of the disease. But everyone else in the world other than myself seems to think these findings have value, so it is OK to leave it in the manuscript. Maybe I just do not think right.

- Perhaps it is due to the idiotic word restrictions imposed by journals, but I think the figures need to be described better:

Fig. 1B: ? microarray studies?

Fig. 1C and 1D: ? RNA? Protein? What are we looking at?

FINALLY, Please do NOT ever send me a manuscript to read with tiny font!!! The easier it is to read a manuscript, the more the reviewer will like it!

1st Revision - authors' response 26 April 2013

Referee #1 (Comments on Novelty/Model System):
Technical quality is high, albeit the data in Fig. 6 are counterintuitive and require many additional controls. Medical impact is medium. Whereas identification of PRL3 as prognostic marker is convincing (high medical impact), the data using PRL3-specific mAbs to target AML cells in nude mice is not convincing (low impact).

Referee #1 (Remarks):
Park et al. investigated the role of PRL3 in AML in relation to the FLT3-ITD mutation. PRL3 expression is upregulated in a significant proportion of FLT3-ITD positive AML patients and cell lines, which is dependent on FLT3 and Src, but not JAK activity. STAT5 is activated concomitantly and PRL3 promoter activity is regulated by STAT5. PRL3 expression in turn is correlated with Jun/AP1 activation and enhanced proliferation. Injection of PRL-3 mAb in mice with engrafted TF-ITD tumors led to a reduction in TF-ITD tumors. Finally, PRL3 expression was identified as a novel prognostic marker independent of other clinical parameters.
This is an interesting paper that reveals the signaling pathway that results in elevated PRL3 expression in FLT3-ITD-positive tumor cells and it describes the identification of PRL3 as a prognostic marker for AML.

We appreciate the important inputs made by the reviewer. The compliment from this reviewer of '*Whereas identification of PRL3 as prognostic marker is convincing (high medical impact)*' is indeed very encouraging to our endeavor on this revision.

Points

1. Fig. 4. Overexpression of PRL3 leads to activation of AP1 in a MEK and JNK dependent manner. It is not evident how PRL3 would mediate this effect. This should be discussed.

We thank and agree with the reviewer for this insightful comment. To address this, we performed additional experiments. Firstly, PRL-3 was overexpressed in a PRL-3 negative cell line-TF-1 (Fig 1C, lane 1) and western blot showed activations of ERK, JNK, and c-Jun. Up-regulation of the phosphorylation of ERK and JNK, concomitant with up-regulation of phosphorylation of-c-Jun were demonstrated (new Fig 4C, b). Secondly, PRL-3 was depleted in two PRL-3 positive AML cell lines (MOLM-14 and TF1-ITD). Depletion of PRL-3 caused down-regulation of the ERK and JNK phosphorylation, accompanied with down-regulation of phosho-c-Jun (new Fig. 4C, a). Thirdly, we used specific MEK inhibitor (U0126) or JNK inhibitor (SP600125) to examine the consequence of ERK or JNK inhibitions in TF-1 cells overexpressing PRL-3 (TF1-PRL-3). We showed that suppression of cell growth after each inhibitor treatment (new Fig. 4D).

It has been reported that phosphatase can activate phosphorylation-mediated signalling pathway through dephosphorylation of inhibitory protein or inhibitory residues (Jaumot & Hancock, 2001; Ory et al, 2003). Thus, we examined several upstream regulators of MEK and JNK pathway to find out the possible link with PRL-3. However, our trial experiments on Ras-Raf family and Akt, upstream regulators of MEK-ERK (Dhillon et al, 2007), did not show any observable reduction on phosphorylation level after overexpression of PRL-3. Since PRL-3 has identified as metastatic protein phosphatase in various cancers, there are several attempts to identify direct substrates of PRL-3 and several PRL-3 interacting proteins were reported by mass spectrometry approach (Ewing et al, 2007), but only very few proteins were proved as interacting molecule of PRL-3 with experimental evidence. And, consistent with our findings, several reports presented that PRL-3 can activate ERK through regulation of upstream activators such as Rho family GTPase (Fiordalisi et al, 2006; Ming et al, 2009), integrin β1 (Peng et al, 2009), or Src (Liang et al, 2007) but underlying mechanism has not fully addressed yet. We have described these in discussion part, page 18, line 5-8.

At this moment, our attempt is not enough to address reviewer's comment, but we are actively working to answer the reviewer's comment, "*how PRL3 would mediate this effect (MAPK activation)*". We appreciate reviewer's insightful scientific comments.

2. The results using PRL3 mAb in the nude TF1-ITD mouse model for AML are puzzling. How can antibodies directed against an intracellular protein affect growth of the target cells in the tumor. The authors apparently have published this unconventional antibody therapy before (discussion,

p.14). These data warrant rigorous controls, as these results are not only unconventional, but also counterintuitive.

We apologize that these important points were not clearly explained in the text and we fully agree with the reviewer that rigorous controls are important since this is an unconventional antibody therapy. In our previous studies, we did use several controls: 1. control antibodies for sham-treatment, 2. cell lines either PRL-3 expressing or PRL-3 non-expressing to show therapeutic response is depending on PRL-3 expression, and 3. control mouse strains (immunodeficient mice: *nude*, *scid*, *muMT* ; immunocompetent mice: C57BL6 wild type, MMTV-PymT spontaneous tumor model) to compare therapeutic efficacies and understand how lymphocytes are involved in this immunotherapy as a Proof-of-Concept. In this current study (Fig. 7, former Fig. 6), we used 3 antibodies to control each other: 1. control IgG antibody for sham-treatment, 2. FLT3 antibody to target *extracellular* FLT3 receptor, and 3. PRL-3 antibody to target *intracellular* PRL-3 phosphatase. We used 3 cell lines: 1. TF-1 cells (non-PRL-3 expression), 2. TF1-ITD cells (both FLT3-ITD and PRL-3 expressions), and 3. TF1-ITD PRL-3 KD cells (reduced PRL-3 expression levels) to study therapeutic efficacies related to target expression levels. If the cancer cells are not expressing target protein (or at low levels), cancer cells will not respond/be affected by the PRL-3 mAb therapy.

Antibodies are traditionally used to target *extracellular* (surface) proteins and have never been used to target *intracellular* proteins because antibodies are generally believed to be too large (~150KD) to enter cells. Indeed, many oncogenic proteins are found within the cell (such as intracellular phosphatases/kinases and transcription factors), leaving a large intracellular treasure of potential cancer-specific therapeutic targets untapped in terms of antibody therapy or vaccination. Since 2005, we have begun to explore if intracellular oncoproteins can be targeted by antibody therapies. We have published several studies since 2008 (*Cancer Biol Ther* 2008; *Sci Transl Med* 2011; *Oncotarget* 2012; *Cancer Res* 2012). Our studies have received encouraging responses from many other labs for collaborations on different 'tumor specific' intracellular oncotargets. Professor Soldano Ferrone (University of Pittsburgh) was commissioned by *Sci Transl Med* to write a Perspective titled *'Hidden Immunotherapy Targets Challenge Dogma'* to accompany our research paper in (*Sci Transl Med* 2011). Actually, since 1978, a substantial body of evidence suggests that antibodies are able to 'penetrate' (most likely via endocytotic pathway) human cells to target intracellular proteins. Alarcon-Segovia D et al. (*Nature*, 1978) described a human immunoglobulin G autoantibody to ribonucleoproteins that could enter viable human lymphocytes using Fc receptors and react with its nuclear antigen. This naturally occurring pathogenic role of autoantibodies in autoimmune diseases indicates that antibody targeting of intracellular oncoproteins might potentially cause cancer cells to self-destruct as well. Although the exact mechanism is yet to be defined, our results so far suggest that antibody can be also used to target *intracellular* oncoproteins for anti-cancer effects in mice models.

Does PRL3 mAb treatment only affect TF1-ITD cells or other tumors as well? For instance, are FLT3-ITD-independent AML cells that do not express elevated levels of PRL3 also affected by PRL3 mAb?

Many thanks for these excellent questions. In our previous studies, we demonstrated that a variety of tumors (regardless of tumor types) expressed PRL-3 antigen was clearly inhibited by PRL-3 antibodies (*Sci Transl Med* 2011). Conversely, if tumors do not express PRL-3 oncotarget, they will not respond/be affected by PRL-3 antibody therapy. Therefore, FLT3-ITD-independent AML cells that do not express elevated levels of PRL-3 will not respond/be affected by PRL3 mAb because an antigen-antibody specific effect must be required in order to achieve therapeutic efficacy.

What is the underlying working mechanism of the mAb treatment?

Several possibilities have been proposed by a well-known immunologist Professor Soldano Ferrone ('Perspective' in *Sci Transl Med* 2011) and by our recent review article *'Awaiting a New Era of Cancer Immunotherapy'* (*Cancer Res* 2012). Three possible mechanisms are proposed as below (A-C):

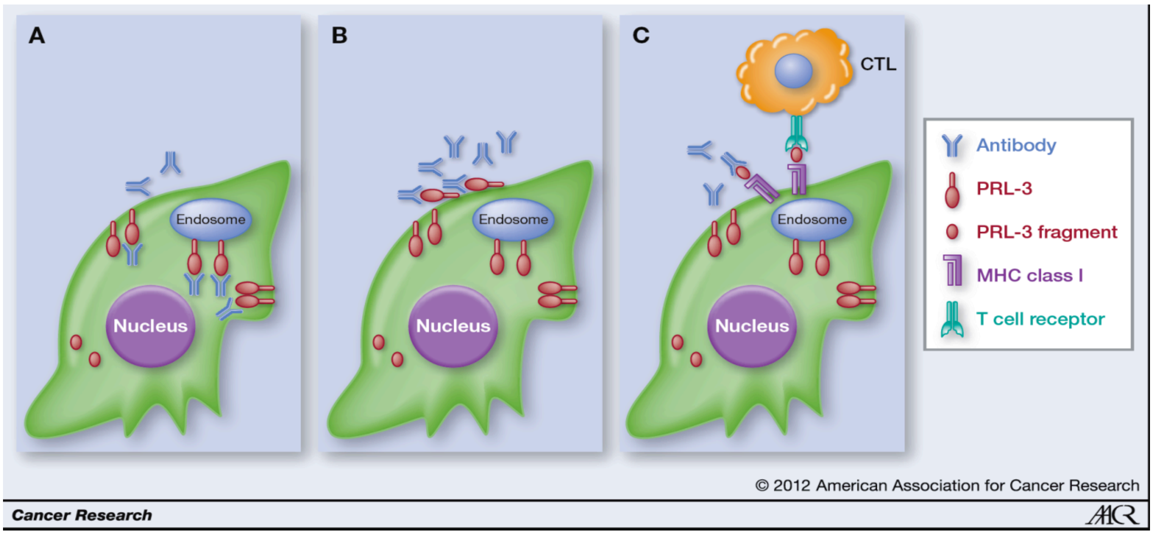

A, antibodies may potentially enter PRL-3–expressing cells, likely via endocytic process (*Cancer Biol Ther*, 2008) to target intracellular PRL-3 and neutralize its function. The interaction of antibodies with the antigens may cause destruction of the cancer cells and lead to apoptosis, a cellular phenomenon previously described for autoantibodies. **B**, some of the intracellular PRL-3 may be externalized and displayed on the surface of cancer cells by unconventional secretion. Binding of antibodies to surface-exposed intracellular proteins may then trigger immune responses such as ADCC to destroy the cancer cells. **C**, proteolytic fragments of intracellular PRL-3 may be presented by MHC class I molecules to attract Cytotoxic T cells (CTLs). It is anticipated that the

combination of several mechanisms may be involved in achieving the final therapeutic consequence of antibodies against intracellular oncoproteins.

Referee #2 (Comments on Novelty/Model System):

Summary of the major findings:
In this study the authors analyze the correlation between the overexpression of PRL-3, a previously identified metastasis-associated tyrosine phosphatase, and FLT3-ITD in AML. By statistical analysis of AML patient data sets the authors show a link between PRL-3 overexpression and FLT3-ITD mutation.
Experiments with AML cell lines show that the constitutive activation of FLT3 enhances PRL-3 expression through the Src-STAT5 signaling pathway and that the up-regulation of PRL-3 induces the expression of c-Jun. However, the study does not provide convincing evidence that PRL-3 acts through a MAPK cascade. More experiments are needed to prove this point.
The authors also analyze effects of PRL-3 on cell proliferation and apoptosis and show that PRL-3 promotes cell proliferation in MTS assays while reducing the number of cells in apoptotic fraction in FACS analysis. The in vivo role of PRL-3 for FLT3-ITD-driven tumor formation was analyzed in a mouse leukemia model by showing that treatment with as monoclonal PRL-3 antibody has anti-tumor effects. The PRL-3 mAb reduces spleen and liver size in mice injected with AML cells comparable to the treatment with FLT3 mAb and reduces AML cell infiltration into the bone marrow in xenograft models. The authors thus demonstrate a potential benefit of PRL-3 immunotherapy. Additionally, statistical evidence is provided showing a correlation of PRL-3 expression with shorter survival in AML patients and revealing PRL-3 as an independent prognostics marker.

Overall, the present study follows up on findings of a previous publication (Zhou et al. 2011), which first showed the correlation between FLT3-ITD mutation and PRL-3 overexpression. Zhou et al. also show that treatment of MOLM14 cells with a PRL-3 inhibitor increases apoptosis which the present study also shows using FACS analysis in figure 5B. The present study presents a significant amount of new data. However, the study has some major flaws. The presented data was not convincing in several cases and some of the conclusions drawn were invalid. The below listed experiments should be repeated or altered as suggested. Especially the study of the in vivo functions of PRL-3 and the potential use of a PRL-3 mAb as a treatment for FLT3-ITD positive patients are of interest from a medical point of view and would merit re-submission of the manuscript.

We are delighted that the reviewer foresees the value of our study of the *in vivo* function of PRL-3 and the potential use of a PRL-3 mAb as a treatment for FLT3-ITD positive patients. Our detailed responses to these insightful inputs are as below.

Comments:

Major issues:

1. The regulation of PRL-3 by FLT-3 analyzed in figure 2A is not convincing in the MOLM-14 cells, as the down-regulation of PRL3 is hardly visible in the western blot analysis. This experiment should be repeated in order to prove that FLT3 activity regulates PRL-3 expression.

We feel sorry that our Fig. 2A was not convincing to show the downregulation of PRL-3 was affected by FLT3 inhibition. To improve this, we repeated the experiment with FLT3 inhibitors (PKC412 and CEP-701) in a dose increment manner. The results now showed that inhibitor treatment clearly reduces FLT3 phosphorylation, concomitant with a decrease in endogenous PRL-3 protein levels in MOLM-14 cells. The improved blots are presented in new Fig. 2A, c-d, which suggest that inhibition of FLT3 activity would downregulate PRL-3 protein expression.

2.1. *In figure 2D the authors analyze the interaction between Src and FLT3 by immunoprecipitation studies in TFI-ITD cells and state that Src an FLT3 do not interact. However, there seems to be some residual Src binding at time point 0, but this hard to tell as the blots in the figure are of poor quality. Negative controls for co-immunoprecipitation experiments and western blots showing protein levels in cellular lysates should be included in the figures.*

We thank and agree with the reviewer's comments on these experiments. We apologize for making the reviewer confused on the physical interaction between Src and FLT3. Although we have repeated the co-IP experiments several times respectively with FLT3, Src, or IgG antibodies using TF1-ITD total cell lysates, we could not obtain a conclusive result to reveal physical interaction between FLT3 and Src, indicating that FLT3 and Src may not directly interact in nature or could due to our technical problems in detecting FLT3 and Src bindings. Therefore, we removed our original Fig. 2D and deleted previous statement *"Src could be an intermediary between FLT3 and STAT5"* and *"interaction of both FLT3-Src and Src-STAT5 pairs was reduced upon CEP-701 mediated interaction of FLT3-ITD"*. Fig. 2 was revised and results were discussed on page 8, line 2-4. We next followed reviewer's kind advices to conduct pull down assay as below.

2.2 The conclusion drawn from the co-immuniprecipitation experiment in figure 2D is not valid. If as claimed Src bridged the interaction between FLT3 and STAT5 then an IP:FLT3 should still pull down Src and STAT5 from cellular lysates. Indirect interactions can be seen in IPs from cellular lysates. The authors should conduct pull-down experiments using purified recombinant proteins in order to analyze whether the interaction between FLT3 and STAT5 is direct or indirect.

Many thanks for the suggestion. Figure 2D was removed. As advised, we performed GST pull-down assay with purified recombinant GST-FLT3 (571-993) protein using either TF1-ITD total cell lysates or using purified recombinant proteins; Src and STAT5. Our trial result (please refer to below Figure) suggests that: **A**. GST alone could not pull down Src or STAT5 from TF1-ITD cell

lysate. **B.** GST-FLT3 (571-993) could pull down Src and STAT5 from TF1-ITD total cell lysates, indicating possible bindings of FLT3, Src, and STAT5. **C**. GST-FLT3 (571-993) could bind to recombinant protein Src much stronger than STAT5 using *in vitro* direct binding assay. At this stage, a conclusive result on their physical interaction was not achieved *in vivo*. Therefore, we did not incorporate this result in the paper. We sincerely seek an understanding from this reviewer.

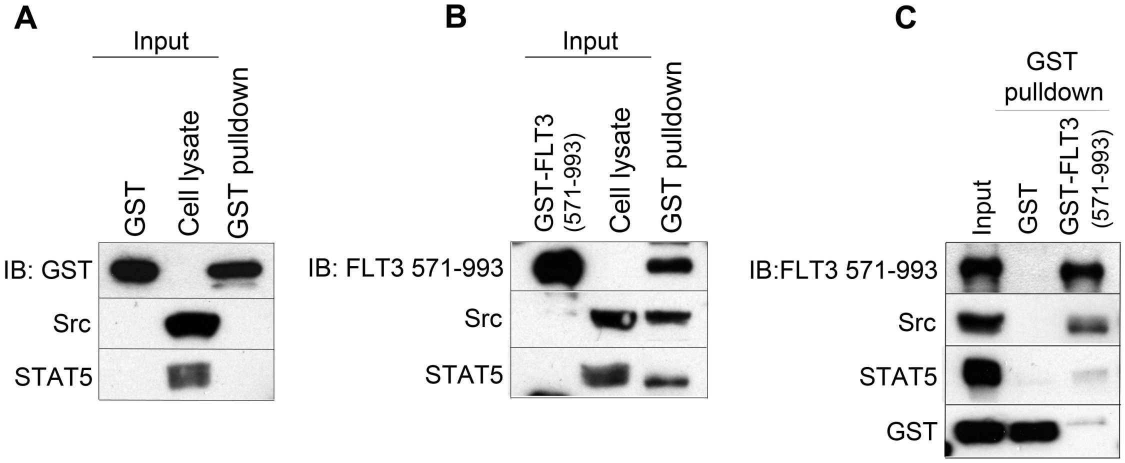

Figure. GST pull-down assay with GST protein alone or GST-tagged FLT3 (571-993) protein *in vitro* TF1-ITD total cell lysate was used for GST pull-down assay with **A.** GST protein could not bind Src or STAT5. **B**. GST-tagged FLT3 (571-993) protein could bind both Src and STAT5. **C**. GST-tagged FLT3 (571-993) protein could bind purified recombinant Src protein stronger than STAT5.

3. In figure 4 the authors make a good case of showing that the expression of PRL-3 induces expression of c-jun. However, they state that PRL-3 acts through a JNK/ERK signaling cascade. They do not provide any prove for this hypothesis. They show that inhibitors of the MAPK JNK and MEK as well as siRNA targeting JNK and ERK result in a decrease in c-Jun phosphorylation. This is to be expected as both JNK and MEK/ERK have been shown to be upstream of c-Jun. The relationship with PRL-3 remains obscure as experiments merely show that c-Jun phosphorylation is mediated by ERK and JNK, which is no new finding.

Many thanks for these important points. To address this, we performed additional experiments. Firstly, PRL-3 was overexpressed in a PRL-3 negative cell line-TF-1 (Fig. 1C, lane 1) and activations of ERK, JNK, and c-Jun were shown by western blot. Up-regulation of the phosphorylation of ERK and JNK, concomitant with up-regulation of phosphorylation of-c-Jun were demonstrated (new Fig. 4C, b). Secondly, PRL-3 was depleted in two PRL-3 positive AML cell lines (MOLM-14 and TF1-ITD). Depletion of PRL-3 caused down-regulation of the ERK and JNK phosphorylation, accompanied with down-regulation of p-c-Jun (new Fig. 4C, a). Thirdly, we used specific MEK inhibitor (U0126) or JNK inhibitor (SP600125) to examine the consequence of ERK or JNK inhibitions in TF-1 cells overexpressing PRL-3 (TF1-PRL-3) (Updated Fig. 4D). These results were incorporated and described from page 10, line 8 to page 11, line 5.

In order to show that PRL-3 mediates up-regulation of c-Jun through JNK/ERK the authors should conduct the following experiments:

- AML cell lines should be depleted of PRL-3 by siRNA and it should be shown that phosphorylation of JNK/ERK is reduced, either by western blotting or by an in vitro kinase assay.

As advised by the reviewer, to understand how PRL-3 would mediate the activation of AP-1 via MEK/ERK and JNK pathways, PRL-3 was depleted in TF1-ITD and MOLM-14 cell lines that highly express endogenous PRL-3 (Fig. 1C, lane 3-4) by performing siRNA knock-down experiments. Depletion of PRL-3 caused down-regulation of the ERK and JNK phosphorylation in 'TF1-ITD PRL-3 KD' and 'MOLM-14 PRL-3 KD' cells when compared to their respective mock-knock downed cell line (new Fig. 4C, a). The down-regulation of ERK and JNK is accompanied with down-regulation of phospho-c-Jun, indicating that PRL-3 may activate c-Jun via ERK and JNK pathways. We added this result in Fig. 4C, a, and explained the results on page 10 (line 16-18). Furthermore, to confirm ERK and JNK are important for PRL-3 to mediate up-regulation of c-Jun, we knocked-down ERK and JNK in TF1-PRL-3 cells. As expected, depletion of ERK or JNK abolished PRL-3 mediated activation of c-Jun (Fig. 4C, c-d).

- Over-expression of PRL-3 in PRL-3 negative cell line (i.e. TFI cells) should induce phosphorylation of JNK and ERK 4.

Thank you for this suggestion! We have overexpressed PRL-3 in TF-1 cell line (TF1-PRL-3), which resulted in upregulation of the phosphorylation level of ERK and JNK in TF1-PRL-3 cells, concomitant with upregulation of phospho-c-Jun (new Fig. 4C, b). These results indicate that PRL-3 activates ERK and JNK pathways to activate c-Jun/AP-1. The result was incorporated in and described on page 10, line 18-20.

A more specific JNK inhibitor should be used in figure 4D in order to prove that the growth advantage of PRL-3 expressing cells relies on activation of the JNK pathway as curcumin effects multiple signaling pathways The conclusion drawn from this experiment is not valid. The authors suggest that PRL-3 is up-stream of c-Jun. However, the experiments presented in 4A and B already show this. The only new result is that curcumin does not affect PRL-3 levels.

To address the growth advantage of PRL-3 actives c-Jun via MEK/ERK and JNK signalling on PRL-3 overexpressing AML cells, we treated TF1-PRL-3 cells with specific MEK/ERK inhibitor (U0126) or JNK inhibitor (SP600125) in addition to curcumin treatment experiment, and assessed cell viability of TF1-PRL-3 cells. As shown in new Fig. 4D (a-b), both inhibitors could respectively reduce cell numbers by around 50% compared to untreated cells. In addition, curcumin treatment could also reduce cell growth in similar manner. The cell growth curves were shown in updated Fig. 4D, and results included on page 11, line 1-5.

Minor issues:

1. Western blot analysis of phospho-FLT3 proving that the FLT3 inhibitors used in figure 2 are either missing or of poor quality. Expression levels of FLT3 in cellular lysates show a high variability at the analyzed time points.

We have repeated western blot analysis with FLT3 inhibitor (PKC412 and CEP-701) treatments. The improved blots were presented in new Fig. 2A, c-d, which show similar expression levels of total FLT3 in cellular lysates, and corresponding decrease of phospho-FLT3 upon inhibitor treatments. In addition, we repeated WB analysis and replaced our previous blots with improved ones (new Fig. 2B, c-d), which show an effect of inhibitor treatment on downstream molecules.

2. A c-fos western blot should be included in figure 4b.

As suggested, new Fig. 4B (b) western blot was included, showing that depletion of PRL-3 in TF1-ITD cells reduced c-Jun but not c-fos expression level.

3. In figure 1 a PRL-3 band clearly visible in FLT3-ITD negative first sample. Thus, 3 out of 12 (25%) FLT3-ITD negative patients appear to show PRL-3 expression. The FLT3 band on far right of FLT3-ITD positive sample appears very faint. In order to strengthen the correlation between FLT3-ITD mutation and PRL-3 expression, an equal number of FLT3-ITD positive and negative AML patient samples should be analyzed.

We agree with the reviewer to include this low PRL-3 expression patient #1 as PRL-3 positive AML. We apologize that we have difficulties to collect equal number of FLT3-ITD positive and FLT3-ITD negative AML patients for this analysis. Since numbers of FLT3-ITD positive patients are lesser than numbers of FLT3-ITD negative patients, we could only obtain these 19 samples from National Hospital of Singapore. Performing qRT-PCR as suggested by reviewer 3, and setting the FLT3-ITD negative patient #1 (lowest PRL-3 expression patient) as a positive reference, we showed 3 out of 12 (25%) FLT3-ITD negative patients and 5 out of 7 (71%) FLT3-ITD positive patients expressed high PRL-3, suggesting that high PRL-3 expression is associated with FLT3-ITD mutation. Data from qRT-PCR was presented in Supporting Information Fig. S1 and explained in page 6, line 9-11. Understanding from this reviewer is greatly appreciated.

4. The effects of PRL-3 overexpression or depletion on AML cells appear marginal in the FACS analysis in figure 5B. The study would benefit from a different apoptosis assay to prove an anti-apoptotic role of PRL-3 more convincingly.

We thank reviewer for pointing out this important comment. TF-1 is a cytokine dependent cell line in terms of cell growth (Lin et al, 2007). In TF1-PRL-3 cells (TF-1 cells overexpressing PRL-3), the

overexpression of PRL-3 likely allows TF-1 cells to activate survival pathways such as AKT and ERK (Jin et al, 2006; Songyang et al, 1997) to allow TF1-PRL-3 cells survive in the absence of cytokines. Consistently, our results show that PRL-3 overexpression could stimulate ERK phosphorylation (Fig. 4C). To further study the anti-apoptotic effect of PRL-3, we performed Annexin-V and 7-AAD staining using TF1-GFP (as control) and TF1-PRL-3 cells, followed by FACS analysis. We observed more cell death in TF1-GFP cells (31% apoptotic cells) than in TF1-PRL-3 cells (6.8% apoptotic cells), supporting that PRL-3 might have a role in anti-apoptotic function in TF-1 cytokine dependent cell line. The data is now shown in Fig. 5C and described on page 11, line 17-23.

Notably, MOLM-14 and MV-11 are cytokine-independent cell lines. Silencing of PRL-3 did not result a substantial increase of apoptotic population as evidenced by the absence of sub-G1 cells (Fig. 5B is now Fig. 6. A-B, b). While depletion of PRL-3 resulted in increased cell numbers in G1 phase but decreased cell numbers in S phase, suggesting that PRL-3 has a role in promoting G1 to S phase transition. To study PRL-3 anti-apoptotic role in these cytokine independent cell lines, Annexin V/7-AAD staining was performed, followed by FACS analyses. We found that depletion of PRL-3 in MOLM-14 and MV4-11 cell lines did not increase apoptotic populations in ‘MOLM-14 PRL-3 KD’ and ‘MV4-11 PRL-3 KD’ cells (new Supporting information Fig. S4). These results support that the role of PRL-3 in these cytokine-independent cell lines is primarily in promoting G1-S phase transition rather than anti-apoptotic function. PRL-3 knock-down might not restore cytokine dependency due to the activation of other compensation pathway(s). Indeed, it has been reported that in AML, several signaling pathway such as RAS, STATs, Bcl-xL, can confer cell transforming activity of cytokine dependent cells to resist apoptosis (Mizuki et al, 2000; Nosaka et al, 1999; Spiekermann et al, 2002).

Referee #3 (Comments on Novelty/Model System):
Important for the reasons given below, along with suggestions for a better model (in the future; these models are not sufficiently developed yet, in my opinion.

We appreciate these thoughtful comments from the reviewer and we have followed his/her advices in preparing our revision. Our detailed responses to these insightful inputs are as below.

Referee #3 (Remarks):

Summary: The treatment of AML (with the exception of APL) has not changed much in the past 40 years, certainly not since this reviewer last cared for an AML patient in 1984. Therefore, it is critical that we develop novel therapies, and PRL3 is certainly an interesting candidate. Here the authors demonstrate an association between FLT3-ITD and PRL3, investigate the mechanism

connecting the two, and demonstrate therapeutic efficacy of PRL3 antibodies. The findings are important in that we desperately need new therapeutic options in AML.

Suggestions for improvement of the manuscript:
There are a number of "minor" but still important corrections that need to be addressed:

- Fig. 1A. I do not understand why anyone does non-quantitative PCR anymore. If the RNA or samples are available, please repeat in a quantitative fashion. If not available, do it next time!

We thank the reviewer for sharing professional experience and fully agree with that "*we desperately need new therapeutic options in AML"*. As advised by the reviewer, we performed qRT-PCR on the 19 AML patients' bone marrows samples (Fig. 1A). Notably, patient #1 (FLT3-ITD negative) also showed low PRL-3 expression level, and we included this patient #1 as PRL-3 positive, and now showed 3 out of 12 (25%) FLT3-ITD negative patients expressed PRL-3, whereas 5 out of 7 (71.4%) FLT3-ITD positive patients expressed PRL-3. This suggests that high PRL-3 expression is associated with FLT3-ITD mutations. Data from q-RT-PCR was presented in Supporting Information Fig S1 and explained in page 6, line 9-11.

- The data supporting the role of STAT5 is much stronger than that of SRC; the authors should soften their statements in the results and discussion.

We agree with reviewer that our statement and conclusion for the role of Src on PRL-3 regulation should be softened since our data was not convincing. To clarify this concern, we deleted our original statements "Src could be an intermediary between FLT3 and STAT5", "Critical role of Src kinase" in discussion. We also removed Fig. 2D 'co-Immunoprecipitation' data which is also not convincing. We amended result section on page 8 (line 2-5) based on updated Fig. 2.

- Fig. 3. It would be desirable (but not absolutely required) to add a STAT5 antibody supershift to the EMSA, and a ChIP assay demonstrating STAT5 binding in cells. The streptavidin-agarose bead assay is a nice addendum to the EMSA.

As advised by the reviewer, we had been trying very hard for this experiment during our revision. Using newly purchased STAT5 antibodies (polyclonal Abs), several attempts on supershift experiments were conducted but the results were still not clear. We reasoned that either an optimized condition was not achieved or our antibodies used were not able to bind STAT5-DNA complexes. At this juncture, we would like to seek this reviewer's understanding of our difficulties.

- Through the manuscript, the authors refer to proliferation (references to Pulikkan and Rangatia page 8, their own Fig. 4C, etc). I believe they are using this term incorrectly, like most of the world. If I understand Fig. 4C correctly, they are measuring cell number, not proliferation. Please correct

throughout the text (and please point out to colleagues that assays of cell number are NOT assays of cell proliferation!).

- Again, in Fig. 5, the authors have completely confused their assays, mixing up cell number, proliferation, and apoptosis. They need to be VERY precise in measuring cell number, proliferation, and apoptosis and be very careful in how they state the results and discussion. It is totally mixed up here, but it can all be easily fixed by being more precise in the labeling and language used. They need to restrain their interpretation of the relative effects of proliferation and apoptosis on cell number unless they perform more assays specific for proliferation and apoptosis.

We are grateful to the reviewer for pointing out our misuses of 'cell numbers, proliferation, and apoptosis' and we feel very sorry about that. Now, we measured and presented with cell numbers for the effects of those inhibitors on cell growth behaviour (new Fig. 4D). Cell numbers were calculated using standard growth curve of each cell line, constructed by absorbance acquired from MTS staining corresponding to cell numbers. We also changed incorrect terms throughout the text and will keep these important points in mind on our future works as well.

As advised by the reviewer, to distinguish "cell number, cell proliferation, and apoptosis" in term of cell growth, we performed Annexin-V staining to indicate apoptotic cells in TF1-GFP verse TF1-PRL-3 cells followed by FACS analysis. We showed more cell death in TF1-GFP cells (31% apoptotic cells) than in TF1-PRL-3 cells (6.8% apoptotic cells), suggesting that PRL-3 plays an anti-apoptotic role in TF-1 cytokine dependent cell line. The data is shown in new Fig. 5C and described in page 11, line 17-23. To study a role of PRL-3 on apoptosis in cytokine independent cell lines, we also performed Annexin-V staining using pairs of 'MOLM-14 verse MOLM-14 PRL-3 KD' cells and 'MV4-11 verse MV4-11 PRL-3 KD' cells. We found that depletion of PRL-3 did not show a increment of apoptotic population in these cytokine independent cell lines (Supporting Information. Fig. 4). Since two cited papers, Pulikkan (Leukemia, 2010) and Rangatia (Oncogene, 2003) describe the role of c-Jun in blocking myeloid differentiation, we cited these papers in the updated text, page 18, line 1.

- Fig. 6. It would be much more desirable to perform these experiments in a better model of FLT3-ITD AML than intravenous injection of transformed cell lines, but the published models using FLT3-ITD alone (either retroviral or knock-in) are not robust. Newer models combining FLT3-ITD with other mutations look more promising, and it will be of great interest to test these models in the future with PRL3 antibody by itself and combined with FLT3-ITD inhibitors.

Many thank for this important input in clinical perspective. As advised, we performed additional animal model to see if we can improve our therapeutic effect when combined PRL-3 mAb with CEP701 inhibitor. Pre-treating mice with FLT3 inhibitor (CEP701), followed by PRL-3 antibody therapy, we found that the CEP701- PRL-3 antibody combined therapies has better therapeutic effects than PRL-3 antibody therapy alone. This is encouraging in our initial trial, however, our data

is not solid and it needs additional experiments to reach a conclusion. At this moment, we sincerely thanks reviewer for this important suggestion from his/her clinical views (Fig. 6 is now Fig. 7).

- Fig. 7. I never understand the value of investigating the prognostic value unless the therapy is related to the gene of interest. Almost all these patients are treated with anthracycline/AraC, so unless response to these drugs is related to PRL3, I do not know how to interpret the data. When PRL3 antibody is used clinically, it would make more sense. This is not natural history of the disease. But everyone else in the world other than myself seems to think these findings have value, so it is OK to leave it in the manuscript. Maybe I just do not think right.

We agree with the reviewer that the prognostic value should reflect the therapy outcome related to the gene of interest. We have a couple of comments here. Firstly, patients treated with *anthracycline/AraC* have different outcomes therefore some gene or network must be influencing the response by the leukaemia cells. Additionally, the exact mechanism of action of *anthracycline/AraC* is not known so it may (or may not) involve PRL-3 or its associated networks. Secondly, the response may be related to PRL-3 directly or indirectly. Nevertheless, the survival curves do indicate that the high levels of PRL-3 can contribute a poorer survival and a less favourable response, which may be of potential clinical use.

In the present study, we have shown that PRL-3 overexpression has an anti-apoptotic effect and enhances cell cycle progression. Therefore, AML patients showing high PRL-3 expression may have higher rate of cancer cell growth, as well as more resistant to apoptosis, than those patients without PRL-3 in their AML cancer cells. The cohort analysis suggests that PRL-3 overexpression contributes to poor prognosis of AML patients, and therefore, targeting or suppressing PRL-3 may be a potential therapeutic approach for PRL-3 overexpressing AML patients. The understanding of this reviewer is sincerely appreciated (Fig. 7 is now Fig. 8).

- Perhaps it is due to the idiotic word restrictions imposed by journals, but I think the figures need to be described better:

Many thanks! We feel sorry for not presenting clearly in our previous figure legends. We have now revised all the Figure legends with a better writing.

Fig. 1B: ? microarray studies?

We apologize that we confused the reviewer. Yes, Fig. 1B is microarray data from four independent AML patient cohorts; Belfast/MILE dataset (Cohort 1), GSE1159, GSE6891, and GSE15434. The data showed a strong association between FLT3-ITD mutation and high PRL-3 expression in a total of 1158 AML patients.

Fig. 1C and 1D: ? RNA? Protein? What are we looking at?

We are very sorry for not being able to describe clearly in the Figure legend. Fig. 1C is the western blot for FLT3 and PRL-3 protein expression levels in four AML cell lines. Fig. 1D is the western blot to show that depletion of FLT3 reduced PRL-3 endogenous protein expression levels.

FINALLY, Please do NOT ever send me a manuscript to read with tiny font!!! The easier it is to read a manuscript, the more the reviewer will like it!

We apologize for the tiny fonts used in our original Figures. To make bigger fonts wherever possible, in this revised manuscript, we have shortened the headings, and deleted unnecessary wordings from the Figures to allow larger font sizes to be used. We hope the reviewer will find them clearer now.

References

Alarcon-Segovia D, Ruiz-Arguelles A, Fishbein E (1978) Antibody to nuclear ribonucleoprotein penetrates live human mononuclear cells through Fc receptors, *Nature* **271**: 67-69

Ferrone S (2011) Hidden immunotherapy targets challenge dogma. *Sci Transl Med* **3:** 99ps38

Dhillon AS, Hagan S, Rath O, Kolch W (2007) MAP kinase signalling pathways in cancer. *Oncogene* **26:** 3279-3290

Ewing RM, Chu P, Elisma F, Li H, Taylor P, Climie S, McBroom-Cerajewski L, Robinson MD, O'Connor L, Li M, Taylor R, Dharsee M, Ho Y, Heilbut A, Moore L, Zhang S, Ornatsky O, Bukhman YV, Ethier M, Sheng Y, Vasilescu J, Abu-Farha M, Lambert JP, Duewel HS, Stewart, II, Kuehl B, Hogue K, Colwill K, Gladwish K, Muskat B, Kinach R, Adams SL, Moran MF, Morin GB, Topaloglou T, Figeys D (2007) Large-scale mapping of human protein-protein interactions by mass spectrometry. *Mol Syst Biol* **3:** 89

Fiordalisi JJ, Keller PJ, Cox AD (2006) PRL tyrosine phosphatases regulate rho family GTPases to promote invasion and motility. *Cancer research* **66:** 3153-3161

Jin A, Kurosu T, Tsuji K, Mizuchi D, Arai A, Fujita H, Hattori M, Minato N, Miura O (2006) BCR/ABL and IL-3 activate Rap1 to stimulate the B-Raf/MEK/Erk and Akt signaling pathways and to regulate proliferation, apoptosis, and adhesion. *Oncogene* **25:** 4332-4340

Liang F, Liang J, Wang WQ, Sun JP, Udho E, Zhang ZY (2007) PRL3 promotes cell invasion and proliferation by down-regulation of Csk leading to Src activation. *J Biol Chem* **282:** 5413-5419

Ming J, Liu N, Gu Y, Qiu X, Wang EH (2009) PRL-3 facilitates angiogenesis and metastasis by increasing ERK phosphorylation and up-regulating the levels and activities of Rho-A/C in lung cancer. *Pathology* **41:** 118-126

Mizuki M, Fenski R, Halfter H, Matsumura I, Schmidt R, Muller C, Gruning W, Kratz-Albers K, Serve S, Steur C, Buchner T, Kienast J, Kanakura Y, Berdel WE, Serve H (2000) Flt3 mutations from patients with acute myeloid leukemia induce transformation of 32D cells mediated by the Ras and STAT5 pathways. *Blood* **96:** 3907-3914

Nosaka T, Kawashima T, Misawa K, Ikuta K, Mui AL, Kitamura T (1999) STAT5 as a molecular regulator of proliferation, differentiation and apoptosis in hematopoietic cells. *The EMBO journal* **18:** 4754-4765

Peng L, Xing X, Li W, Qu L, Meng L, Lian S, Jiang B, Wu J, Shou C (2009) PRL-3 promotes the motility, invasion, and metastasis of LoVo colon cancer cells through PRL-3-integrin beta1-ERK1/2 and-MMP2 signaling. *Mol Cancer* **8:** 110

Songyang Z, Baltimore D, Cantley LC, Kaplan DR, Franke TF (1997) Interleukin 3-dependent survival by the Akt protein kinase. *Proceedings of the National Academy of Sciences of the United States of America* **94:** 11345-11350

Spiekermann K, Pau M, Schwab R, Schmieja K, Franzrahe S, Hiddemann W (2002) Constitutive activation of STAT3 and STAT5 is induced by leukemic fusion proteins with protein tyrosine kinase activity and is sufficient for transformation of hematopoietic precursor cells. *Experimental hematology* **30:** 262-271

2nd Editorial Decision 11 June 2013

Thank you for the submission of your revised manuscript to EMBO Molecular Medicine. We have now received the enclosed reports from the Reviewers 1 and 3 (Reviewer 2 was not available) that were asked to re-assess it. As you will see, since the Reviewers are now supportive and you have also satisfactorily addressed Reviewer 2's concerns, I am pleased to inform you that we will be able to accept your manuscript pending the following final amendments:

1) As per our Author Guidelines, the description of all reported data that includes statistical testing must state the name of the statistical test used to generate error bars and P values, the number (n) of independent experiments underlying each data point (not replicate measures of one sample), and the actual P value for each test (not merely 'significant' or 'P < 0.05').

2) Please correct the mistake in Fig. 4C as indicated by Reviewer 1

Finally, I suggest you take note of Reviewer 3's very sound advice in the future!

Please submit your revised manuscript within two weeks. I look forward to seeing a revised form of your manuscript as soon as possible.

***** Reviewer's comments *****

Referee #1 (Remarks):

Minor point: Fig. 4C. c-Jun should read p-c-Jun in all panels.

Referee #3 (Remarks):

The authors have done a good job responding to all of the reviewers. I only have a few "editorial" comments:

1. I look forward to when PRL-3 antibodies are used clinically in humans.

2. I urge the authors to continue in the future to try to understand the mechanisms of how PRL-3 works (what are the direct targets and pathways), as well as how the antibody works. Both are important and interesting questions.

3. The authors misunderstood my remarks about fonts. While I am pleased that authors increased the sizes of the fonts in the figures, I was also referring to the size of the fonts used in the text of the manuscript. Assuming you really want me to read it, why use 10 point font? Next time please increase the font size of the TEXT ! (Hint:

try 12 point).

Also, it would be useful to label the figures as well ("Figure

1", "Figure 2", etc) on the same side of the page as the figure.

Again, the easier it is for the reviewer to read, the happier we will be...

2nd Revision - authors' response 18 June 2013

Response to Editorial comments:

1) As per our Author Guidelines, the description of all reported data that includes statistical testing must state the name of the statistical test used to generate error bars and P values, the number (n) of independent experiments underlying each data point (not replicate measures of one sample), and the actual P value for each test (not merely 'significant' or 'P < 0.05').

Thank you for your guidance. We amended our manuscript according to Author Guidelines to show the name of the statistical test to generate error bars and actual P values. When actual p-value was low ($p < 1x\ 10^{-3}$), we used '$p < 0.001$' where it was applicable. Three independent experiments were performed. Please see below table.

	Amended on manuscript
Fig. 1B. Microarray data<br><br>*Chi-square test*	In **Fig. 1B legend** on page 37<br><br>a. $p = 0.001$<br><br>b-d. $p < 0.001$
Fig. 3F, a. Q-RT-PCR<br><br>*Student's t-test*	In **Fig. 3F legend** on page 39<br><br>***$p < 0.001$ for TF1-ITD<br><br>**$p = 0.011$ for MOLM-14<br><br>*$p = 0.038$ for MV4-11
Fig. 5C. FACS analysis<br><br>*Student's t-test*	In **Fig. 5C legend** on page 40<br><br>***$p = 0.0012$

Fig. 7<br><br>7A. *Student's t-test*<br><br><br>7B. *Student's t-test*<br><br><br>7C. *Kaplan-Meier analysis*	In **Fig. 7 legend** on page 41<br><br>*$p < 0.001$; **$p = 0.00121$<br><br><br>*$p < 0.001$<br><br><br>$p < 0.001$
Fig. 8<br><br>*Kaplan-Meier survival analysis*	In **Fig. 8**<br><br>**A**. $p = 0.028$ **B**. $p < 0.001$ **C**. $p = 0.025$
Table 1. *Cox-regression analysis*	<u>*Age*</u><br><br>Univariate, $p < 0.001$<br><br>Multivariate, p < 0.001<br><br><u>*Cytogenetic risk*</u><br><br>Intermediate, univariate, p < 0.001<br><br>Intermediate, multivariate, p = 0.001<br><br>Adverse, univariate, p < 0.001<br><br>Adverse, multivariate, p < 0.001

© EMBO
