# Supplementary material for: Oncogenic roles of PRL-3 in FLT3-ITD induced acute myeloid leukaemia
Source: EMBO Mol Med. 2013 Aug 8;5(9):1351–66. doi: 10.1002/emmm.201202183 (PMC3799491; doi:10.1002/emmm.201202183)
Supplement: Supplementary file 2 [file emmm0005-1351-SD2.pdf]

Supporting Information

Oncogenic Roles of PRL-3 in FLT3-ITD Induced Acute Myeloid Leukemia

Jung Eun Park, Hiu Fung Yuen, Jian Biao Zhou, Abdul Qader O. Al-aidaroos, Ke Guo, Peter J Valk, Shu Dong Zhang, Wee Joo Chng, Cheng William Hong, Ken Mills and Qi Zeng

Table of Contents

Supporting Information

Figure S1. Quantitative real-time PCR analysis for AML patients' bone marrow samples

: High PRL-3 mRNA expression was associated with AML patients with FLT3-ITD mutation.

Figure S2. STAT5A and STAT5B protein expression levels with different reporter vectors.

Figure S3. AP-SEAP activity assay in DLD-1 and HCT116 cells

: PRL-3 overexpression activates AP-1 activity.

Figure S4. Annexin-V and 7-AAD staining with MOLM-14 and MV4-11 cells

: Depletion of PRL-3 shows no substantial increment in apoptotic population in two cytokine independent cell lines, MOLM-14 and MV4-11.

Supporting Information Figure 1

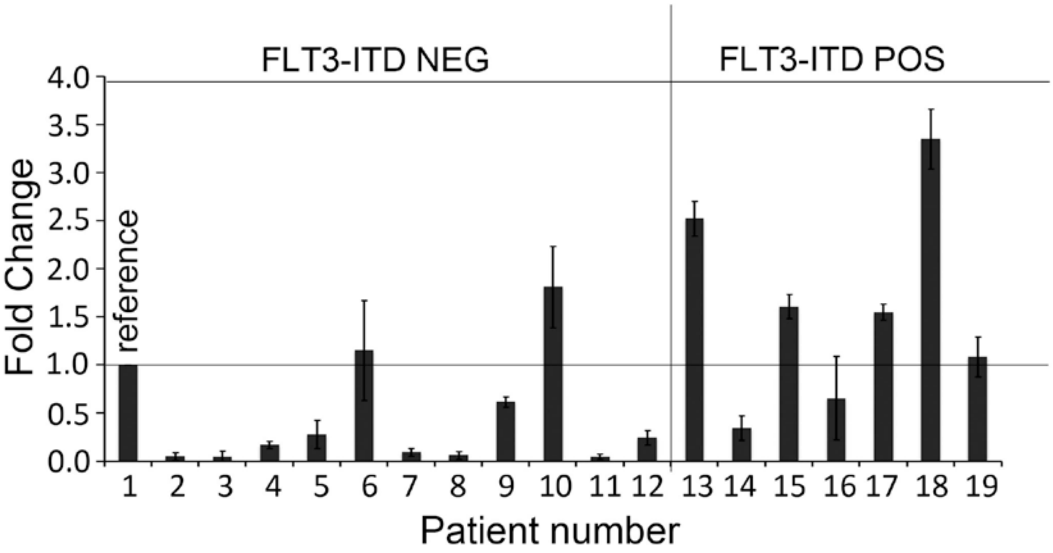

Figure S1. High PRL-3 mRNA expression was associated with AML patients with FLT3-ITD mutation.

PRL-3 mRNA levels were assessed in 19 AML patients' bone marrow samples by quantitative real-time PCR (qRT-PCR) analysis. Up-regulation of PRL-3 mRNA was shown in patient #1, #6, and #10 with FLT3-ITD negative mutation (NEG, n=12), and in patient #13, #15, #17, #18, and #19 with FLT3-ITD positive mutation (POS, n=7). For quantification of relative PRL-3 mRNA level, patient #1 was set as 1 for reference. Error bars represent the mean ± SD from three independent experiments. NEG, negative; POS, positive

Supporting Information Figure 2

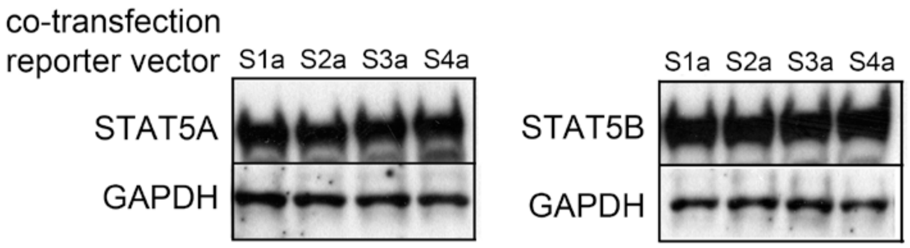

Figure S2. Similar STAT5A and STAT5B protein expression levels were detected in TF-1 cells expressing different reporter constructs.

For the luciferase reporter assay, pCMV6-STAT5A or pCMV6-STAT5B expression vector was co-transfected respectively with pGL-Luc-S1a, -S1b, -S1c, -or -S1d constructs in TF-1 cells. Western blots showed similar expression levels of STAT5A and STAT5B at all conditions.

Supporting Information Figure 3

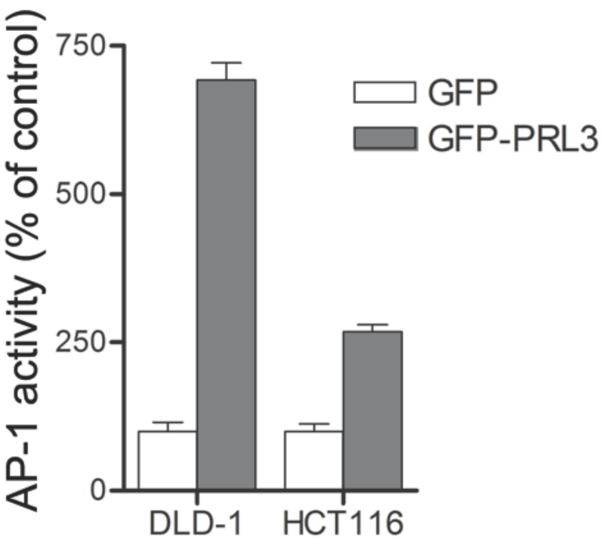

Figure S3. PRL-3 overexpression activates AP-1 activity. Activation of AP-1 activity was examined using two solid tumor cell lines, DLD-1 and HCT116. Each cell line was transiently co-transfected with AP-1 SEAP reporter vector along with either GFP or GFP-PRL-3 expression vector. Overexpression of PRL-3 led to a 6.5-fold and >2.5-fold increase in AP-1 activity in DLD-1 and HCT116 cells, respectively. Error bars represent the mean ± SD from three independent experiments.

Supporting Information Figure 4

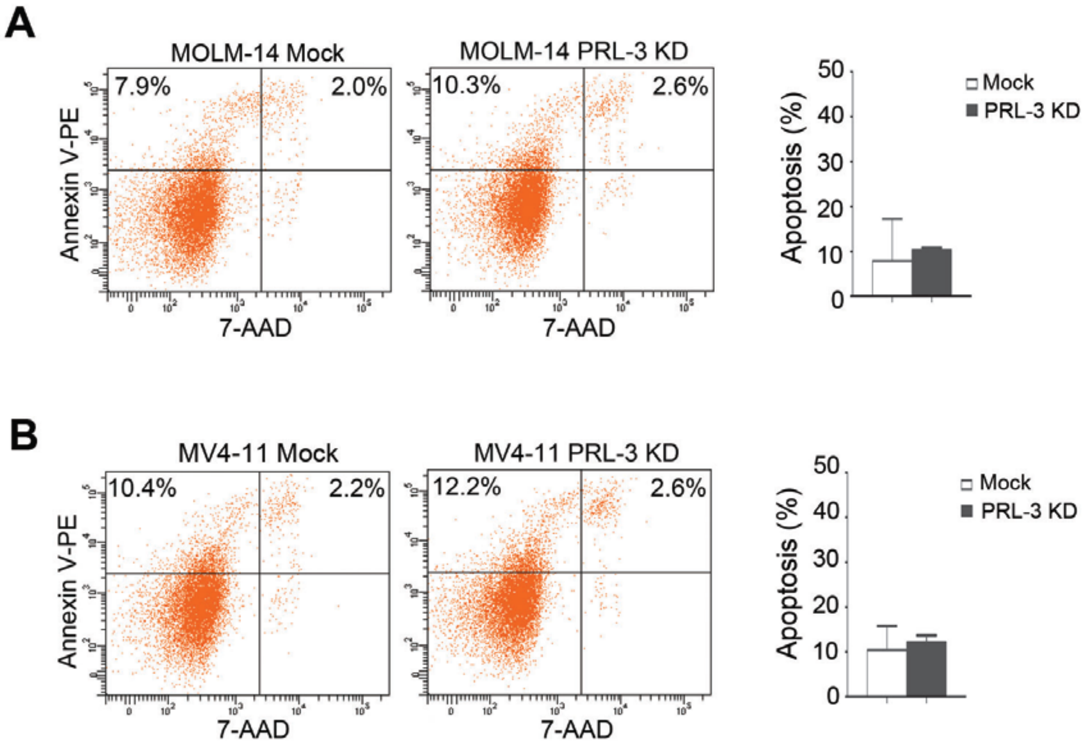

Figure S4. Depletion of PRL-3 shows no substantial increment in apoptotic population in two cytokine independent cell lines, MOLM-14 and MV4-11.

Apoptotic activity of PRL-3 was assessed by Annexin-V and 7-AAD staining, followed by FACS analysis. The populations of Annexin V-positive cells are shown on top left corner of each panel. MOLM-14 and MV4-11 mock-knock down cells showed around 7.9% and 10.4% of Annexin-V positive cells, and PRL-3 depleted MOLM-14 and MV4-11 cells (MOLM-14 PRL-3 KD and MV4-11 PRL-3 KD) showed ~10.3% and ~12.6% of apoptotic population.

A. *Left panel*, flow cytometry analysis of annexin-V- and 7-AAD-stained MOLM-14 and MOLM-14 PRL-3-KD cells after 48 hr culture. *Right panel*, quantitation of annexin-V-positive apoptotic population in three independent experiments (mean ±SD, n=3).

B. *Left panel*, flow cytometry analysis of annexin-V- and 7-AAD-stained MV4-11 and MV4-11 PRL-3-KD cells after 48 hr culture. *Right panel*, quantitation of annexin-V-positive apoptotic population in three independent experiments (mean ±SD, n=3).
